# Comparing clinical and echocardiographic outcomes following valve-sparing versus transannular patch repair of tetralogy of Fallot: a systematic review and meta-analysis

**DOI:** 10.1093/icvts/ivae124

**Published:** 2024-06-26

**Authors:** Russell Seth Martins, Asad Saulat Fatimi, Omar Mahmud, Saleha Qureshi, Muhammad Taha Nasim, Sehar Salim Virani, Aimen Tameezuddin, Fatima Yasin, Mahim Akmal Malik

**Affiliations:** Division of Thoracic Surgery, Department of Surgery, Hackensack Meridian School of Medicine and Hackensack Meridian Health Network, Edison, NJ, USA; Medical College, Aga Khan University, Karachi, Pakistan; Medical College, Aga Khan University, Karachi, Pakistan; Medical College, Aga Khan University, Karachi, Pakistan; Medical College, Aga Khan University, Karachi, Pakistan; Department of Surgery, Aga Khan University, Karachi, Pakistan; Medical College, Ziauddin University, Karachi, Pakistan; Medical College, Aga Khan University, Karachi, Pakistan; Department of Cardiac Surgery, Rawalpindi Institute of Cardiology, Rawalpindi, Pakistan

**Keywords:** Valve-sparing, Transannular patch, Tetralogy of Fallot, Meta-analysis, Clinical outcomes, Echocardiographic outcomes

## Abstract

**OBJECTIVES:**

Transannular patch (TAP) repair of tetralogy of Fallot (ToF)relieves right ventricular tract obstruction but may lead to pulmonary regurgitation. Valve-sparing (VS) procedures can avoid this situation, but there is a potential for residual pulmonary stenosis. Our goal was to evaluate clinical and echocardiographic outcomes of TAP and VS repair for ToF.

**METHODS:**

A systematic search of the PubMed, Embase, Scopus, Cochrane Central Register of Controlled Trials and Web of Science databases was carried out to identify articles comparing conventional TAP repair and VS repair for ToF. Random-effects models were used to perform meta-analyses of the clinical and echocardiographic outcomes.

**RESULTS:**

Forty studies were included in this meta-analysis with data on 11 723 participants (TAP: 6171; VS: 5045). Participants who underwent a VS procedure experienced a significantly lower cardiopulmonary bypass time [mean difference (MD): −14.97; 95% confidence interval (CI): −22.54, −7.41], shorter ventilation duration (MD: −15.33; 95% CI: −30.20, −0.46) and shorter lengths of both intensive care unit (ICU) (MD: −0.67; 95% CI: −1.29, −0.06) and hospital stays (MD: −2.30; 95% CI: [−4.08, −0.52). There was also a lower risk of mortality [risk ratio: 0.40; 95% CI: (0.27, 0.60) and pulmonary regurgitation [risk ratio: 0.35; 95% CI: (0.26, 0.46)] associated with the VS group. Most other clinical and echocardiographic outcomes were comparable in the 2 groups.

**CONCLUSIONS:**

This meta-analysis confirms the well-established increased risk of pulmonary insufficiency following TAP repair while also demonstrating that VS repairs are associated with several improved clinical outcomes. Continued research can identify the criteria for adopting a VS approach as opposed to a traditional TAP repair.

## Graphical Abstract

**Figure:**
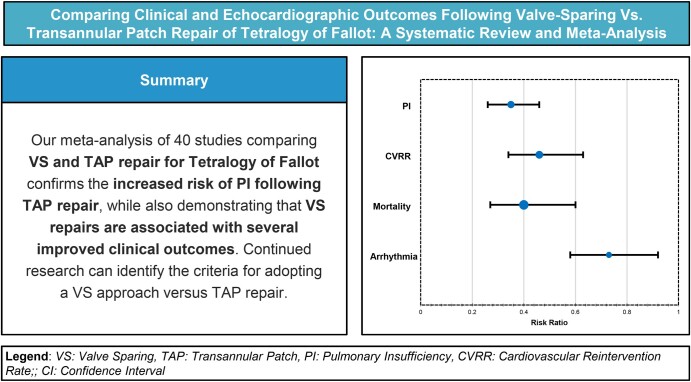

## INTRODUCTION

Tetralogy of Fallot (ToF) affects roughly 5 in every 10000 live births and is the most common cyanotic congenital heart defect, accounting for 7% to 10% of all congenital heart defects [1, 2]. The conventional surgical approach involves relieving right ventricular outflow tract obstruction (RVOTO) by sacrificing the competence of the pulmonary valve (PV) and inserting a transannular patch (TAP). This approach adequately restores RVOT patency and is associated with a low risk of restenosis [3]. However, the ill effects of subsequent pulmonary insufficiency (PI) and corresponding right ventricle (RV) volume overload on long-term cardiac health led to the introduction of valve-sparing (VS) procedures. These enable the continued growth and function of patients’ PVs but have raised concerns of inadequate correction of pulmonary stenosis, which could cause pathologically high RV afterload and high reoperation/reintervention rates [4, 5].

Although numerous studies have been conducted to assess the relative effectiveness and safety of VS and TAP procedures, there is as yet no consensus regarding which of these approaches is superior. The goal of this study was to systematically review all of the existing literature comparing clinical and echocardiographic outcomes of patients after VS versus TAP surgery for ToF.

## METHODS

This systematic review followed the Preferred Reporting Items for Systematic Reviews and Meta Analyses (PRISMA) guidelines (Supplementary Material, Section 1) [6] . All data were obtained from published articles, precluding the need for institutional review board approval. This review was preregistered with PROSPERO (CRD42022326324), and authorship was determined using a published rubric [7].

### Search strategy

Systematic searches of the PubMed, Embase, Scopus, Cochrane Central Register of Controlled Trials and Web of Science databases were conducted on 27 April 2024 (Supplementary Material, Section 2). Additional studies were identified via the references of previous reviews and from available grey literature, such as conference abstracts. Observational and randomized studies were considered eligible for inclusion irrespective of year of publication.

### Study selection criteria

The inclusion criteria were as follows:

1. Observational or randomized studies
2. Studies reporting data per the following PICO (Population, Intervention, Control, Outcomes) question:
  - **P:** Patients with ToF
  - **I:** VS repair
  - **C:** TAP repair
  - **O:** Postoperative clinical and echocardiographic outcomes

The exclusion criteria were as follows:

1. Uncontrolled studies such as case reports or case series
2. Studies in which patients had ToF with pulmonary atresia or complete atrioventricular canal defect
3. Studies without accessible English language full texts

### Screening process

Two reviewers (O.M. and A.S.F.) independently screened the titles and abstracts of all articles retrieved during the search process. Full-text review was conducted in a similar manner to determine the final set of included articles. Conflicts were resolved by a third reviewer (R.S.M.). Corresponding authors were contacted via email to solicit relevant data that were missing from manuscripts/supplements.

### Data extraction

All data were independently extracted by 2 reviewers (A.M., S.Q., M.T.N. or F.Y.) with discrepancies resolved by a third reviewer (A.S.F. or S.S.V.). Study and patient characteristics were abstracted, along with relevant clinical and echocardiographic variables and corresponding time-intervals of measurement. To avoid pooling the same patients multiple times, studies with cohorts sampled from the same population (i.e. at the same hospitals during overlapping time periods) were identified and a single study was pooled; those with propensity matched cohorts were given preference, followed by those with larger sample sizes (Supplementary Material, Section 3).

Given that some data in 1 study, Lv *et al.*, were not reported numerically but as graphs, WebplotDigitizer version 4.5 was used to derive numerical values for relevant data points [8, 9]. It is a freely available tool that has been validated previously in the literature and enables accurate data extraction with excellent inter-rater correlations (> 95%) [10, 11]. Extraction of graphical data was performed by 2 reviewers (A.S.F. and M.T.N.). The average agreement was >99%.

### Approximating means and standard deviations for non-parametrically distributed data

Non-parametrically distributed continuous data were extracted as sample medians with ranges or interquartile ranges. We used the methods of Wan *et al.* [12], which allowed us to approximate means and standard deviations from these data to enable meta-analyses (Supplementary Material, Section 4). These methods are more generalizable for scenarios with small sample sizes *n*, given that other methods do not incorporate *n* into calculations. Non-parametrically distributed clinical outcome data from 2 studies, Jiang *et al.* and Sasson *et al.*, were excluded because of extreme outliers that precluded accurate approximations [13, 14].

### Continuity corrections for zero-event studies

Where 0 events in a cohort were reported for dichotomous variables, a continuity correction was used to include the study data in the analysis. For studies that reported 0 events in 1 arm (single zero-event studies), a continuity correction of 0.5 was added to the arm automatically by Review Manager version 5.4.1 (The Cochrane Collaboration, Copenhagen, Denmark). For studies that reported 0 events in both arms (double zero-event studies), the Carter estimator outlined by Wei *et al.* [15] was utilized whereby 1 was added to the number of events in both arms, and 2 was added to the total number of participants in both arms. This step was deemed necessary because these results are not necessarily non-informative, and their exclusion may introduce estimation bias to the overall effect size [16, 17].

### Quality assessment (risk of bias) and certainty of evidence assessment

Two reviewers (S.Q. and S.S.V.) independently evaluated risk of bias in the included studies using the Risk of Bias in Non-Randomized Studies of Intervention tool for observational studies [18]. The certainty of evidence for each outcome was determined using the GRADE approach and recorded independently by 2 reviewers (A.S.F. and O.M.) via the GRADE Pro Software (McMaster University and Evidence Prime Inc, Hamilton, Ontario, Canada) [19]. Conflicts were resolved by a third reviewer (R.S.M.). Publication bias was assessed using funnel plots generated for each outcome with more than 10 studies in keeping with the *Cochrane Handbook* [20].

### Statistical analyses

All meta-analyses were performed using RevMan v5.4.1 (The Cochrane Collaboration, Copenhagen, Denmark). In cases with multiple outcome measurements during follow-up, the most recent was pooled. Meta-regression was performed with median duration of follow-up as a study level covariate [via Stata v17 (StataCorp LLC, College Station, TX, USA)]. Wherever provided, propensity score matched data were used for the analysis instead of full cohort data. Continuous outcomes were all measured using the same scales and were pooled as mean differences (MDs) with 95% confidence intervals (CIs). Dichotomous outcome measures were pooled as risk ratios (RRs) with 95% CIs. Forest plots were generated for all meta-analysed outcomes. Sensitivity analysis was used to assess the impact of each study on the overall effect size.

Given that only observational data were available, efforts were made to incorporate baseline comparability of patient cohorts into the analysis. All analyses were stratified into subgroups based on study level comparisons of pulmonary valve annulus *z*-scores (PVA *z*-scores) across arms. That is, studies were subgrouped into (i) studies in which preoperative PVA *z*-scores were statistically different between cohorts—all such studies were found to have higher preoperative PVA *z*-scores in their VS cohorts, (ii) studies with no significant difference and (iii) studies for which there was no information to conclude whether or not a statistically significant difference was present. This method was deemed appropriate because, like these comparisons of average preoperative PVA *z*-scores across cohorts, the results of random-effects meta-analysis are also group level statistics, and because individual participant data are not available in the literature.

Given the differences in the designs and characteristics of the included studies, inverse variance and Cochran–Mantel–Haenszel random effects models were used to perform meta-analyses of continuous and dichotomous outcomes, respectively. The proportion of heterogeneity in our results is measured and reported as the Higgins I^2^ statistic, with >50% being considered moderate, and >75% being considered substantial. All *P*-values were two-sided, and a *P*-value < 0.05 was considered significant for all analyses.

## RESULTS

### Literature search

**Figure 1:**
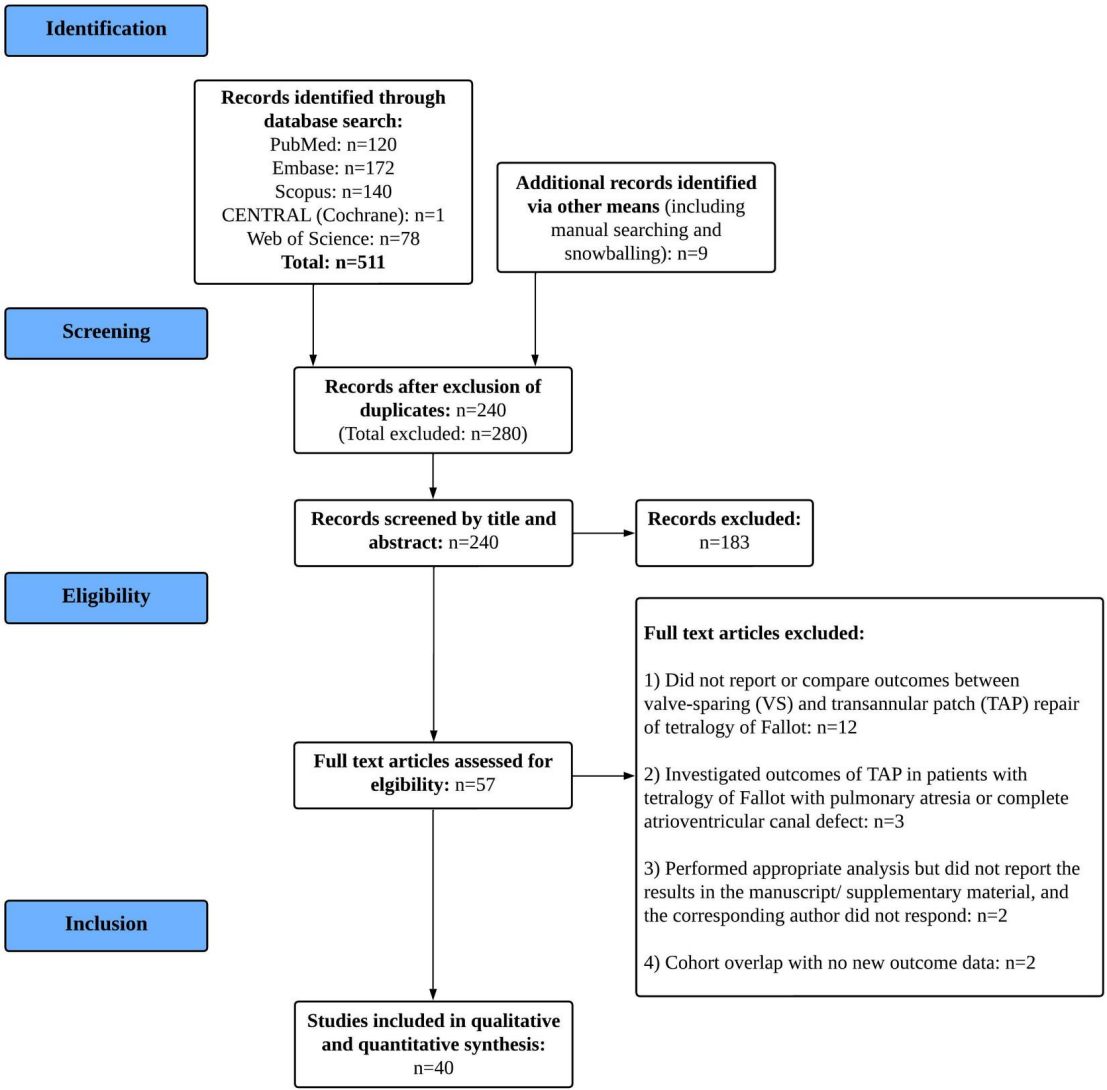
PRISMA flow chart delineating study selection. PRISMA: Preferred Reporting Items for Systematic Reviews and Meta-Analyses.

Our search strategy yielded 240 unique articles. Forty cohort studies (37 retrospective, 3 prospective), which comprised 37 non-overlapping cohorts, were included in the review [5, 8, 13, 14, 21–56]. Seven of the included retrospective cohort studies had performed propensity score matched analyses. A diagrammatic breakdown of our literature search process is presented in Fig. 1.

### Study and participant characteristics

The 40 included studies included 11,723 patients (TAP: 6,171; VS: 5,045). Most studies were conducted in North America (15/40; 37.5%), followed by Asia (17/40; 32.4%), Europe (7/40; 17.5%) and Africa (1/40; 2.5%). Nine had cohorts with comparable preoperative PVA *z*-scores. Supplementary Material, Section 5 includes the study (Table A) and preoperative participant characteristics (Table B) of all the included studies.

### Meta-Analysed Results

Summarized results of all the intraoperative and postoperative clinical and echocardiographic variables included in the meta-analyses and the associated heterogeneity statistics are present in Tables 1 and 2, with individual forest plots available in Supplementary Material, Section 6. In all analyses, subgroups A, B and C represent subgroups of studies in which the baseline PVA *z*-score was significantly lower in TAP patients, comparable in TAP and VS groups, or not characterized.

**Table 1:** Clinical outcomes after valve-sparing versus transannular patch repair for tetralogy of Fallot

Variable		Number of studies				RR/MD [95% CI] for VS group				*P*-value for subgroup differences (A vs B)	τ^2^	I^2^ (%)	Certainty of evidence
		Overall	Subgroups			Overall	Subgroups						
			A	B	C		A	B	C				
Continuous variables													
Intraoperative	XCT (min)	17	11	5	1	−**4.33 [**−**8.14,** −**0.52]**	−**9.12 [**−**14.50,** −**3.74]**	1.67 [−2.77, 6.12]	6.67 [−2.60, 15.93]	**0.002**	46.38	85	Very low
	CBPT (min)	15	11	4		−**14.97 [**−**22.54,** −**7.41]**	−**21.26 [**−**32.28,** −**10.23]**	1.64 [−5.27, 8.55]		**<0.001**	186.66	93	Very low
Early postoperative (in-hospital)	Length of ICU stay (days)	10	5	4	1	−**0.67 [**−**1.29,** −**0.06]**	−**1.28 [**−**2.38,** −**0.18]**	0.09 [−0.77, 0.95]	−**0.67 [**−**0.89,** −**0.45]**	**0.05**	0.85	93	Very low
	Ventilation duration (h)	9	5	4		−**15.33 [**−**30.20,** −**0.46]**	−20.86 [−42.85, 1.12]	−5.61 [−15.49, 4.27]		0.22	488.78	99	Very low
	Length of hospital stay (days)	9	5	4		−**2.30 [**−**4.08,** −**0.52]**	−1.92 [−4.56, 0.71]	−**2.59 [**−**5.02,** −**0.16]**		0.71	6.17	92	Low
	Postoperative inotropic support duration (h)	4	3	1		−17.64 [−53.42, 18.13]	−26.50 [−60.00, 6.99]	7.10 [−0.76, 14.96]		0.06	1280.36	98	Very low
Dichotomous variables													
Early postoperative (in-hospital)	Arrhythmia	10	8	1	1	**0.73 [0.58, 0.92]**	**0.73 [0.57, 0.93]**	0.84 [0.39, 1.80]	0.17 [0.01, 3.01]	0.74	0.00	0	Very low
	ECMO	6	4	1	1	**0.42 [0.28, 0.64]**	**0.44 [0.29, 0.66]**	0.33 [0.01, 8.16]	0.13 [0.01, 2.38]	0.87	0.00	0	Moderate
	Pleural effusion	4	3		1	**0.47 [0.24, 0.92]**	0.51 [0.26, 1.00]		0.13 [0.01, 2.38]		0.00	0	Low
	AKI	5	5			**0.29 [0.16, 0.51]**	**0.29 [0.16, 0.51]**				0.00	0	Low
	Neurologic complications	4	4			0.60 [0.15, 2.45]	0.60 [0.15, 2.45]				0.52	25	Very low
	Chylothorax	3	3			0.34 [0.09, 1.22]	0.34 [0.09, 1.22]				0.22	16	Low
	Pericardial effusion	2	2			0.22 [0.03, 1.52]	0.22 [0.03, 1.52]				0.93	47	Very low
Late postoperative (follow-up)	Cardiovascular reintervention rate	21	14	5	2	**0.46 [0.34, 0.63]**	**0.38 [0.30, 0.49]**	0.91 [0.23, 3.58]	0.56 [0.30, 1.05]	0.22	0.14	33	Low
	Deaths during follow-up (overall)	18	10	5	3	**0.40 [0.27, 0.60]**	**0.52 [0.29, 0.95]**	0.38 [0.11, 1.35]	**0.32 [0.12, 0.85]**	0.65	0.00	0	Low

Subgroup A: Studies in which preoperative pulmonary valve annulus *z*-scores were reported to be statistically different between cohorts.

Subgroup B: Studies in which preoperative pulmonary valve annulus *z*-scores were reported to have no significant difference between groups.

Subgroup C: Studies in which a *P*-value for this comparison was not provided, and there was no information to conclude whether or not a statistically significant difference was present.

Values in **bold** represent statistically significant results (*P* < 0.05).

AKI: acute kidney injury; CBPT: cardiopulmonary bypass time; CI: confidence interval; ICU: intensive care unit; ECMO: extracorporeal membrane oxygenation; MD: mean difference; RR: risk ratio; VS: valve-sparing; XCT: aortic cross-clamp time.

**Table 2:** Echocardiographic outcomes after valve-sparing versus transannular patch repair for tetralogy of Fallot

Variable		Number of studies				RR/MD [95% CI] for VS group				*P*-value for subgroup differences (A vs. B)	τ^2^	I^2^ (%)	Certainty of evidence
		Overall	Subgroups			Overall	Subgroups						
			A	B	C		A	B	C				
Continuous variables													
Postoperative	RVOT pressure gradient (mmHg)	13	12	1	0	0.69 [−1.45, 2.83]	0.36 [−1.82, 2.54]	5.40 [−0.71, 11.51]		0.13	8.20	68	Very low
	PVA Z-score	6	4	1	1	−0.23 [−1.05, 0.6]	0.04 [−0.33, 0.41]	−**1.70 [**−**2.14,** −**1.26]**	**0.50 [0.16, 0.84]**	**<0.001**	0.84	93	Very low
	RV/LV pressure ratio	7	7	0	0	−0.00 [−0.08, 0.07]	−0.00 [−0.08, 0.07]				0.01	90	Very low
	Maximum RVOT velocity (m/s)	2	1	0	1	−0.25 [−0.55, 0.05]	−0.10 [−0.70, 0.50]		−0.30 [−0.65, 0.05]		0	0	Very low
	RV/LV size ratio	2	1	0	1	−**0.25 [**−**0.31,** −**0.19]**	−**0.27 [**−**0.34,** −**0.20]**		−**0.25 [**−**0.31,** −**0.19]**	0.33	0	0	Moderate
Dichotomous variables													
Postoperative	Pulmonic insufficiency	24	15	7	2	**0.35 [0.26, 0.46]**	**0.39 [0.29, 0.53]**	**0.24 [0.08, 0.70]**	0.12 [0.00, 11.64]	0.37	0.37	90	High
	Residual RVOT obstruction/stenosis	5	5	3	0	1.01 [0.53, 1.95]	0.84 [0.43, 1.67]	2.36 [0.30, 18.80]		0.36	0.32	45	Very low
	Tricuspid regurgitation	7	6	1	0	1.16 [0.59, 2.30]	1.02 [0.45, 2.34]	1.84 [0.72, 4.72]		0.36	0.32	42	Very low
	RV systolic dysfunction	3	1	2	0	0.83 [0.54, 1.28]	0.95 [0.21, 4.32]	0.82 [0.52, 1.29]		0.86	0	0	Low
	RVOT Dilation	2	1	1	0	0.24 [0, 28.7]	**0.05 [0.01, 0.36]**	0.99 [0.65, 1.49]		**0.004**	11.37	96	Very low

Subgroup A: Studies in which preoperative pulmonary valve annulus *z*-scores were reported to be statistically different between cohorts.

Subgroup B: Studies in which preoperative pulmonary valve annulus *z*-scores were reported to have no significant difference between groups.

Subgroup C: Studies in which a *P*-value for this comparison was not provided and there was no information to conclude whether or not a statistically significant difference was present.

Values in **bold** represent statistically significant results (*P* < 0.05).

CI: confidence interval; MD: mean difference; RR: risk ratio; VS: valve-sparing.

#### Intraoperative variables

Aortic cross-clamp time (XCT, minutes) (Supplementary Material, Fig. 6.1.1) was significantly longer in TAP compared to VS in subgroup A [MD: −9.12 (−14.50, −3.74)] and overall [MD: −4.33 (−8.14, −0.52)]. Cardiopulmonary bypass time (CPBT, minutes) (Supplementary Material, Fig. 6.1.2; Table 1) was significantly longer in TAP patients in subgroup A [MD: −21.26 (−32.28, −10.23)] and overall [MD: −14.97 (−22.54, −7.41)].

#### Postoperative clinical outcomes

Cardiovascular reintervention rates (Supplementary Material, Fig. 6.2.1) were significantly greater after TAP versus VS overall [RR: 0.46 (0.34, 0.63)] and subgroup A [RR: 0.38 (0.30, 0.49)], but not in subgroup B (Table 1). The results of the test for differences between subgroups A and B were insignificant (*P* = 0.27).

The incidence of overall mortality (Supplementary Material, Fig. 6.2.2) was significantly lower after VS surgery versus TAP overall [RR: 0.40 (0.27, 0.60)] and subgroups A [RR: 0.52 (0.29, 0.95)] and C [RR: 0.32 (0.12, 0.85)] but not in subgroup B. Only Blais *et al.*, Ono *et al.* and Smith *et al*. independently detected a survival advantage after VS repair [5, 26, 51]. Sensitivity analyses showed that the significance of the overall result was not influenced by the inclusion of any particular study or of double zero-event studies. Subgroup B did not show a significant difference, but the result of the test for differences between subgroups A and B was also insignificant (*P* = 0.65).

Length of stay in the intensive care unit (ICU) (days) (Supplementary Material, Fig. 6.2.3) was significantly shorter after VS overall [MD: −1.28 (−2.38, −0.18)]. A similar result was obtained in subgroup A [−0.67 (−1.29, −0.06)]. Four studies were pooled in subgroup B and reported widely varying results: 1 study identified a significantly higher mean duration after VS, 1 after TAP and 2 studies identified no significant difference.

Ventilation duration (hours) (Supplementary Material, Fig. 6.2.4) was significantly longer after TAP repair overall [MD: −15.33 (−30.20, −0.46)]. Neither subgroup A nor B achieved significant results.

Rates of arrhythmia (Supplementary Material, Fig. 6.2.5) were significantly greater in the TAP group versus the VS group overall [RR: 0.73 (0.58, 0.92)] and subgroup A [RR: 0.73 (0.57, 0.93)] but not subgroup B (although this only had 1 study).

Length of hospital stay (days) (Supplementary Material, Fig. 6.2.6) was significantly longer after TAP overall [MD: −2.30 (−4.08, −0.52)] and in subgroup B [MD: −2.59 (−5.02, −0.16)]. Although subgroup A did not achieve significance, the test of differences between subgroups A and B was also insignificant (*P* = 0.71).

The incidence of extracorporeal membrane oxygenation (Supplementary Material, Fig. 6.2.7) was higher in the TAP group versus the VS group overall [RR: 0.42 (0.28, 0.64)] and in subgroup A [RR: 0.44 (0.29, 0.66)]. Although there were no significant differences between subgroups (*P* = 0.87), the sole study in subgroup B did not report a significant difference.

Duration of inotropic support (h) (Supplementary Material, Fig. 6.2.8) did not differ overall between the VS and TAP groups [MD: −17.64 (−53.42, 18.13)], and the included studies were heterogeneous.

Pleural effusion (defined as pleural drainage for greater than or equal to 2 days) (Supplementary Material, Fig. 6.2.9) occurred more commonly after TAP overall [RR: 0.47 (0.24, 0.92)].

Acute kidney injury (Supplementary Material, Fig. 6.2.10) occurred more frequently after TAP [RR: 0.29 (0.16, 0.51)], whereas the incidence of neurologic complications [RR: 0.60 (0.15, 2.45); Supplementary Material, Fig. 6.2.11], chylothorax [RR: 0.34 (0.09, 1.22); Supplementary Material, Fig. 6.2.12] and pericardial effusion [RR: 0.22 (0.03, 1.52); Supplementary Material, Fig. 6.2.13] was similar in both the VS and the TAP groups.

#### Postoperative echocardiographic outcomes

The incidence of moderate or severe PI (Supplementary Material, Fig. 6.3.1; Table 2) was significantly higher after TAP overall [RR: 0.35 (0.26, 0.46)] in subgroup A [RR: 0.39 (0.29, 0.53)] and subgroup B [RR: 0.24 (0.08, 0.70)]. Testing for differences between the pooled RRs of subgroups A and B did not achieve significance (*P* = 0.37). Additionally, although the data could not be pooled in the meta-analysis, Taksaudom *et al.* [54] performed a multivariable propensity score matched analysis that showed TAP to be an independent predictor of PI [hazard ratio: 2.51 (1.23, 5.13)].

The RVOT pressure gradient (mmHg) (Supplementary Material, Fig. 6.3.2) did not differ overall across VS and TAP [MD: 0.69 (−1.45, 2.83)] or in subgroups A [0.36 (−1.82, 2.54)] or B [MD: 5.40 (−0.71, 11.51)]. However, subgroup B consisted of only a single study.

Postoperative PVA *z*-score (Supplementary Material, Fig. 6.3.3) was not different overall [MD: −0.23 (−1.05, 0.60)] or in subgroup A [MD: 0.04 (−0.33, 0.41)]. Subgroup B, a single study (*n* = 58), showed significantly larger postoperative PVA *z*-scores in the TAP group [MD: −1.70 (−2.14, −1.26)].

Incidence of residual RVOTO or stenosis (Supplementary Material, Fig. 6.3.4) was similar after VS and TAP overall [RR: 1.01 (0.53, 1.95)], as well as subgroups A [RR: 0.84 (0.43, 1.67)] and B [RR: 2.36 (0.30, 18.80)]. Although the data could not be pooled in the meta-analysis, Taksaudom *et al.* [54] performed a multivariable propensity score matched analysis in which TAP showed a protective effect against moderate-severe RVOTO in the long term compared with the VS group [hazard ratio: 0.14 (0.02, 0.90)].

The pooled incidence of tricuspid valve regurgitation (TVR) (Supplementary Material, Fig. 6.3.5) was similar in VS and TAP patients overall [RR: 1.94 (1.03, 3.65)], as well as in both subgroups A [RR: 1.02 (0.45, 2.34)] and B [RR: 1.84 (0.72, 4.72)], which consisted of a single study (*n* = 43).

Pooled RV/LV pressure ratio (Supplementary Material, Fig. 6.3.6) was similar after VS and TAP procedures [MD: −0.00 (−0.08, 0.07)]. All 7 studies had significantly smaller PVA *z*-scores in their TAP cohorts.

RV systolic dysfunction (Supplementary Material, Fig. 5.3.7) occurred at similar rates after VS and TAP overall [RR: 0.83 (0.54, 1.28)] and in subgroups A [RR: 0.95 (0.21, 4.32)] and B [RR: 0.82 (0.52, 1.29)]. However, only 3 studies were pooled in this result.

Additional results with <3 pooled studies are reported in Table 2 and Supplementary Material, Section 6.

### Meta-regression analyses

Meta-regression was performed to identify if any of the observed heterogeneity in the pooled rates of PI (24 studies pooled), cardiac reintervention (21 studies pooled) and mortality (18 studies pooled) was attributable to different durations of follow-up. The utility of this analysis was limited by the small number of included studies, and the results were not informative (Supplementary Material, Section 7).

### Risk of Bias Assessment

Twelve studies had low overall risk of bias, 13 studies had moderate risk of bias and 15 studies had serious risk of bias. None had critical risk of bias. All studies with serious risk of bias did not appropriately control for important confounders and baseline differences between arms (such as PVA *z*-score). All studies deemed to have a moderate risk of bias were also down-rated due to confounding, with 1 study additionally being down-rated because of bias due to missing data. Complete risk of bias assessment results are available in Supplementary Material, Section 8.

### Certainty of evidence assessment

The overall certainty of evidence was low. One of the included outcomes was assessed to have a high certainty of evidence, 2 were assessed to have a moderate certainty of evidence, 7 were assessed to have a low certainty of evidence, whereas the remaining 15 had a very low certainty of evidence. The quality of evidence was predominantly down-rated due to the moderate/severe risk of bias of the studies being pooled. Although certainty of evidence was assessed for overall outcomes, subgroup analysis was done to offset the risk of bias in the meta-analysis where applicable. The domain-wise certainty of evidence assessment for all outcomes is available in Supplementary Material, Section 9.

## DISCUSSION

This systematic review and meta-analysis compared perioperative clinical and echocardiographic outcomes after VS versus TAP repair of ToF. Our results show that patients selected for VS repair were significantly less likely to develop PI. In addition, no significant postoperative differences were seen in residual RVOTO, RVOT pressure gradients, PVA *z*-scores, TVR, RV systolic dysfunction and RV/LV pressure ratios. We found that VS operations were performed with shorter intraoperative CBPTs and XCTs. Moreover, VS operations were associated with lower postoperative rates of cardiovascular reintervention, arrhythmia, mortality, extracorporeal membrane oxygenation, pleural effusion and AKI and shorter lengths of ventilation, ICU admission and hospital stay. Our findings suggest that a VS operation successfully decreases the risk of PI while enabling the safe repair of ToF in selected patients.

Refined surgical techniques and improved orchestration of perioperative care have lowered postoperative mortality to <1.5% and enabled >95% survival to adulthood of patients with repaired ToF [5, 33]. However, TAP repair results in PV incompetence and regurgitation. Subsequent RV volume overload incites maladaptive dilation and remodelling that eventually leads to right ventricular systolic dysfunction [49]. Pathological RV:LV pressure and volume ratios may also compromise LV function. These patients often require pulmonary valve replacements (PVR) with multiple redo operations to mitigate the onset of complications including diminished functional capacity, poor exercise tolerance, arrhythmia, heart failure and sudden cardiac death [33, 48]. However, the optimal timing of PVR remains unknown, and the reverse remodelling it stimulates does not completely normalize the ventricles [49, 57, 58]. Techniques to reconstruct the PV in conjunction with TAP repair have evolved and limit postoperative PI but are associated with early failure and a lack of growth potential [59, 60]. Consequently, the focus has shifted towards preserving a competent native PV that grows with the rest of the heart [59].

Our pooled analysis shows that patients selected for VS operations were significantly less likely to develop PI [RR: 0.35 (0.26, 0.46)] and risk difference [RD]: −0.43 (−0.52, −0.34), and no study individually reported results to the contrary. Crucially, this trend persisted in the subgroup of studies in which baseline PVA *z*-scores were not significantly different between VS and TAP groups [RR: 0.24 (0.08, 0.70), RD: −0.33 (−0.50, −0.17)], suggesting the ability of VS procedures to prevent PI versus TAP in patients with comparable baseline PVA size. The large absolute effect size, indicated by the RD, indicates the clinical significance of this result.

However, the risk of significant PI after VS is evidently not zero [40]. Patients with underdeveloped or dysplastic PVs are inherently more prone to PI despite VS procedures which attempt to enable the functionality of such valves [40]. This observation is particularly associated with anomalous morphologies like bicuspid PV or extremely small annulus size [61]. In other cases, disproportionate growth of the annulus or leaflets (possibly due to the degeneration of biomaterial patches when used to augment valve leaflets) or excessive balloon dilatation of the annulus may compromise PV competence [30, 31]. Given the strong prognosis of most patients after TAP repair and improvements in the safety and efficacy of percutaneous valve insertion, it has been argued that TAP repair is adequate despite the presence of PI and will become even more favourable with time [23, 42, 62]. However, the absolute reduction in PI risk in our results was large and can be reliably achieved in many patients (such as those with tricuspid PVs that are not severely hypoplastic) [59]. In addition, even in cases in which VS does not prevent PI, evidence has shown that the degree of PI after a VS operation may improve with time but remains stationary or worsens after a TAP [59]. A VS procedure may also confer a prognostic advantage because, although not detected in earlier studies, multiple newer studies with large cohorts and long-term follow-up have identified superior long-term survival in patients treated with VS repair [5, 26, 63, 64]. Thus, VS procedures are successful in decreasing the risk of PI and may have valuable functional and survival benefits.

No significant difference in the rates of TVR was seen between VS and TAP patients [51]. Recent studies have questioned if RV dilation due to severe PI contributes to TVR in repaired ToF, and studies in adults who underwent repair in childhood have shown poor correlation between RV and right atrium volumes, tricuspid annular dilatation and the presence of significant TVR [65]. On the other hand, TVR itself, when compounded by significant PI, may result in RV dilation and subsequent complications in patients followed after PV replacement [59, 66]. In addition, significant TVR in patients with repaired TOF has been associated with atrial fibrillation independent of PI severity [67, 68]. Our results suggest that patients develop TVR at similar rates after VS versus TAP. More studies are needed to understand the relationship between TVR and RV function [42].

A major concern with VS repair has been that of residual or recurrent RVOT stenosis. These stenoses lead to pathologically high afterload in the right circuit and can be due to an inherently small annulus or insufficient application of the technique applied to repair the PV such as leaflet commissurotomy, balloon dilatation of the annulus, infundibulum muscle bundle resection and supravalvular patching [31, 59]. Echocardiographic measurement of RVOT obstruction, RVOT pressure gradients and RV:LV pressure and volume ratios can identify residual stenosis [32]. Similarly, post-repair intraoperative PVA *z*-scores are useful to assess the adequacy of RVOTO correction and can predict the longevity of competent PVs after VS repair [24, 46]. We found no significant difference in rates of RVOTO [RR: 1.01 (0.53, 1.95)], RVOT pressure gradients [MD: 0.69 (−1.45, 2.83)], RV:LV pressure ratios [MD: −0.00 (−0.08, 0.07)] or PVA *z*-scores [MD: −0.23 (−1.05, 0.60)] after VS versus TAP. Moreover, subgroup analysis of 3 studies in which TAP and VS groups had comparable baseline PVA *z*-scores also showed no significant differences in RVOTO [RR: 2.36 (0.30, 18.80)]. However, the only study with comparable baseline status and data on post-operative PVA *z*-scores showed that they were higher after TAP [MD: −1.70 (−2.14, −1.26)]. Thus, although our results suggest that VS procedures are not followed by increased rates of RVOTO, there is sparse and mixed evidence when comparable patients are compared across VS versus TAP procedures. High RV:LV pressure ratios (with cut-offs in the literature ranging from 0.85 to 0.7) are considered indicative of insufficient correction of RVOT stenosis and are associated with poorer outcomes [45, 69]. Some patients have persistently high RVOT gradients postoperatively that may represent subvalvular stenosis that required further resection than was performed [46]. Unlike direct measurements of pressure made intraoperatively to determine relief of RVOTO, intraoperative transoesophageal echocardiography *z*-scores (<−3.2) better identify the need for further subpulmonary resection [46]. Thus, it has been suggested that a thorough assessment be performed to determine if RVOTO has been sufficiently relieved, if additional resection is required or if a conversion to TAP is indicated [24, 46, 70]. However, it is difficult to accurately delineate those patients who will experience less morbidity and reintervention with VS repair (despite related residual RVOT stenosis) from those who will benefit more from a TAP, albeit with PI. We conclude that VS repair does not always lead to higher rates of residual RVOT stenosis, that studies reporting these results are heterogeneous, and that the literature advocates for a more nuanced interpretation of results based on study populations.

We found significantly lower cardiovascular reintervention rates after VS operations [RR: 0.46 (0.34, 0.63)]. Only 2 studies independently reported significant differences: Jiang *et al.* found higher reintervention rates in the TAP group, whereas Hofferberth *et al.* reported increased reintervention rates in the VS cohort [13, 40]. As discussed previously, it has been suggested that improved operative techniques promote the growth and patency of the RVOT and that PV can help to avoid the need for intervention [71]. However, the results of these studies do not generalize to all patients. For example, Hofferberth *et al.* [40] only included patients receiving intraoperative balloon dilatation of stenotic pulmonary valves and found that their patients often developed PI despite a VS approach. Indeed, a challenge with the use of intraoperative balloon dilatation is to avoid irreversible damage by over-stretching the annulus, potentially leading to reintervention requirements [60]. Interestingly, it has also been suggested that the reintervention risk after TAP because of long-term PI may be underestimated by studies due to inadequate follow-up [72]. In fact, comparing reintervention rates over 30 years of follow-up in a propensity score matched cohort, Blais *et al.* [5] found lower rates of reintervention in their VS cohort that persisted even when considering only patients with significant residual pulmonary stenosis. Although it was limited to just 5 studies, there was no significant difference in our subgroup analysis of reintervention rates in patients with comparable baseline PVA *z*-scores. It is possible that successful VS repair in patients selected on the basis of certain factors, such as birth weight and age (<55 days), does not increase the risk of cardiac reinterventions. Moreover, there is evidence that VS may in fact have lower reintervention rates in some subpopulations, but that this may be indicative of disease severity and patient characteristics rather than higher durability of VS repair. Clinicians must continue to individualize their approach to each patient’s specific anatomy.

Our meta-analysis showed that patients were likely to be placed on cardiopulmonary bypass for significantly shorter durations after VS procedures [MD: −14.97 (−22.54, −7.41)]. Subgroup analysis showed that this situation was attributable primarily to studies in which significantly lower preoperative PVA *z*-scores were seen in TAP groups [MD: −21.26 (−32.28, −10.23)] but not those in which TAP and VS cohorts had similar baseline PV sizes [MD: 1.64 (−5.27, 8.55)]. In the pooled analysis of XCT, significantly longer XCTs were seen during TAP [MD: −4.33 (−8.14, −0.52)], once again attributable to the subgroup with significantly smaller preoperative PVA *z*-scores in TAP patients [MD: −9.12 (−14.50, −3.74)]. The non-pulsatile blood flow through a cardiopulmonary bypass circuit lacking endothelium is thought to induce deleterious complement activation and endothelial dysfunction in patients' circulations [73]. Similarly, prolonged aortic cross-clamping can lead to ischaemia–reperfusion injury of clinical consequence [74]. Furthermore, global longitudinal strain—a marker of cardiac function that is more sensitive than the ejection fraction [75, 76]—is abnormally low after ToF repair and does not completely normalize over time (with respect to age matched controls) [49]. This observation has been attributed in part to duration of cardiopulmonary bypass and myocardial ischaemia [49]. As with cardiovascular reintervention rates, the shorter cardiopulmonary bypass time and XCT in VS operations seen in these results may reflect greater disease severity in patients selected for TAP who required longer and more complex operations rather than a technical advantage of a VS operation over TAP.

Analysis of postoperative clinical outcomes showed that patients undergoing VS repair were less likely to die [RR: 0.40 (0.27, 0.60)], have pleural effusion [RR: 0.61 (0.20, 1.88)], and have acute kidney injury [RR: 0.30 (0.09, 0.97)]. Lower rates of arrhythmia and extracorporeal membrane oxygenation—which is associated with postoperative mortality—were also seen with VS surgery [47]. Furthermore, ventilation duration [MD: −15.33 (−30.20, −04.60)] and lengths of both ICU [MD: −0.67 (−1.29, −0.06)] and hospital [MD: −2.30 (−4.08, −0.52)] stays were significantly shorter in the VS group [47]. Notably, hospital stay was also significantly shorter in the subgroup analysis of patients with comparable baseline PVA *z*-scores across groups. However, the impact of several factors associated with postoperative outcomes after ToF repair is not captured in our analysis. It has been shown that the presence of additional non-modifiable factors such as anomalies outside the heart, prematurity, low birth weight and non-elective surgery (indicative of an emergency patient condition) predict greater morbidity and logistical burden in ToF repair [72]. Thus, our results suggest that those patients who are identified as candidates for VS repair tend to experience a smoother postoperative course, in addition to potential decreases in psychological and financial harm to parents. However, these results must be considered in the context that ToF repair is already established to be extremely safe.

Our meta-analysis is limited by the small cohort sizes from retrospective observational studies. The generalizability of our analysis is also constrained by the non-trivial heterogeneity in some results. Moreover, echocardiography is operator-dependent, and cardiac MRI data, which are considered the “gold” standard for non-invasive characterization of cardiac function and viability, were not reported by any of the included studies [77]. In particular, the selection of the technique of the operation is determined by the inherent size of the PV annulus, whereby a cohort of patients will be selected out of receiving a VS procedure as a result. It is also worth noting that some studies lacked information regarding procedural variations for each of TAP and VS repairs, thereby precluding this analysis from capturing potential confounding. This finding limits the applicability of these results, and the evidence reported by the included studies is generally specific to granularly defined patient cohorts and operative techniques and must be interpreted accordingly. Our overall results are thus indicative of how patients fare, given that they were selected for and underwent VS versus TAP procedures but do not inform causality. We performed subgroup analyses based on the comparability of baseline PVA *z*-scores, with the intention of providing results with greater baseline comparability of patients. However, group level subanalyses in this vein are not a substitute for original studies that analyse the outcomes of matched or randomized patients. As such, this meta-analysis of observational studies largely serves to describe outcomes given current practices and to generate hypotheses rather than to inform causality. Although it is understood that randomized studies may be infeasible given the nature of ToF repair, it may be stated that an important finding of this systematic review is the dearth of observational studies in the literature with comparable or matched cohorts.

In conclusion, our results suggest that VS repairs succeed in limiting the PI associated with the use of TAP in what may be a safer operative approach in eligible patients. However, whether or not this translates to superior long-term health and survival is determined by multiple factors, such as the exact phenotype and severity of ToF in individual patients, the timing and staging of initial primary repair and additional elements that remain to be fully characterized. Thus, continued research to identify precise morphological criteria and progress in the operative and technological aspects of ToF repair will more clearly distinguish patients in whom VS procedures are superior from those in whom a TAP is more appropriate.

## Supplementary Material

ivae124_Supplementary_Data

## Data Availability

All data in this systematic review and meta-analysis were sourced from studies that have been previously published.

## Notes

Presented at the 8th World Congress of Pediatric Cardiology and Cardiac Surgery in Washington, DC, USA on August 29th, 2023.

## ABBREVIATIONS

CI: Confidence intervals
ICU: Intensive care unit
MD: Mean difference
PI: Pulmonary insufficiency
PV: Pulmonary valve
PVA: Pulmonary valve annulus
RR: Risk ratio
RV: Right ventricle
RVOTO: Right ventricular outflow tract obstruction
TAP: Transannular patch
ToF: Tetralogy of Fallot
TVR: Tricuspid valve regurgitation
VS: Valve-sparing
XCT: Aortic cross-clamp time
