# Supplementary material for: Comparing clinical and echocardiographic outcomes following valve-sparing versus transannular patch repair of tetralogy of Fallot: a systematic review and meta-analysis
Source: Interdiscip Cardiovasc Thorac Surg. 2024 Jun 26;39(1):ivae124. doi: 10.1093/icvts/ivae124 (PMC11283307; doi:10.1093/icvts/ivae124)
Supplement: ivae124_Supplementary_Data [file ivae124_supplementary_data.docx]

**Comparing Clinical and Echocardiographic Outcomes Following Valve-Sparing Versus Transannular Patch Repair of Tetralogy of Fallot: A Systematic Review and Meta-Analysis**

**Supplementary Material**

*Supplementary Section 1:* PRISMA Checklist (2020)

*Supplementary Section 2:* Complete Search Strings

*Supplementary Section 3:* Studies Excluded Due to Overlapping Cohorts

*Supplementary Section 4:* Parametric Estimations of Non-Parametric Data

*Supplementary Section 5:* Study and Participant Characteristics

*Supplementary Section 6:* Forest Plots

*Supplementary Section 7:* Bubble Plots for Meta-Regression

*Supplementary Section 8:* Risk of Bias Assessment

*Supplementary Section 9:* GRADE Certainty of Evidence Assessment

**Section 1: PRISMA Checklist (2020)**

| **Section and Topic** | **Item #** | **Checklist item** | **Reported on Page #** |
| --- | --- | --- | --- |
| **TITLE** | | |  |
| Title | 1 | Identify the report as a systematic review. | 1 |
| **ABSTRACT** | | |  |
| Abstract | 2 | See the PRISMA 2020 for Abstracts checklist. | 2 |
| **INTRODUCTION** | | |  |
| Rationale | 3 | Describe the rationale for the review in the context of existing knowledge. | 4 |
| Objectives | 4 | Provide an explicit statement of the objective(s) or question(s) the review addresses. | 4 |
| **METHODS** | | |  |
| Eligibility criteria | 5 | Specify the inclusion and exclusion criteria for the review and how studies were grouped for the syntheses. | 5-6 |
| Information sources | 6 | Specify all databases, registers, websites, organisations, reference lists and other sources searched or consulted to identify studies. Specify the date when each source was last searched or consulted. | 5 |
| Search strategy | 7 | Present the full search strategies for all databases, registers and websites, including any filters and limits used. | 5, Supplementary Section 2 |
| Selection process | 8 | Specify the methods used to decide whether a study met the inclusion criteria of the review, including how many reviewers screened each record and each report retrieved, whether they worked independently, and if applicable, details of automation tools used in the process. | 5-6 |
| Data collection process | 9 | Specify the methods used to collect data from reports, including how many reviewers collected data from each report, whether they worked independently, any processes for obtaining or confirming data from study investigators, and if applicable, details of automation tools used in the process. | 6-8 |
| Data items | 10a | List and define all outcomes for which data were sought. Specify whether all results that were compatible with each outcome domain in each study were sought (e.g. for all measures, time points, analyses), and if not, the methods used to decide which results to collect. | 6-7 |
|  | 10b | List and define all other variables for which data were sought (e.g. participant and intervention characteristics, funding sources). Describe any assumptions made about any missing or unclear information. | 6-7 |
| Study risk of bias assessment | 11 | Specify the methods used to assess risk of bias in the included studies, including details of the tool(s) used, how many reviewers assessed each study and whether they worked independently, and if applicable, details of automation tools used in the process. | 8 |
| Effect measures | 12 | Specify for each outcome the effect measure(s) (e.g. risk ratio, mean difference) used in the synthesis or presentation of results. | 9 |
| Synthesis methods | 13a | Describe the processes used to decide which studies were eligible for each synthesis (e.g. tabulating the study intervention characteristics and comparing against the planned groups for each synthesis (item #5)). | 6-7, Supplementary Section 5 |
|  | 13b | Describe any methods required to prepare the data for presentation or synthesis, such as handling of missing summary statistics, or data conversions. | 6-7 |
|  | 13c | Describe any methods used to tabulate or visually display results of individual studies and syntheses. | 9 |
|  | 13d | Describe any methods used to synthesize results and provide a rationale for the choice(s). If meta-analysis was performed, describe the model(s), method(s) to identify the presence and extent of statistical heterogeneity, and software package(s) used. | 8-9 |
|  | 13e | Describe any methods used to explore possible causes of heterogeneity among study results (e.g. subgroup analysis, meta-regression). | 8-9 |
|  | 13f | Describe any sensitivity analyses conducted to assess robustness of the synthesized results. | 8-9 |
| Reporting bias assessment | 14 | Describe any methods used to assess risk of bias due to missing results in a synthesis (arising from reporting biases). | 8 |
| Certainty assessment | 15 | Describe any methods used to assess certainty (or confidence) in the body of evidence for an outcome. | 8 |
| **RESULTS** | | |  |
| Study selection | 16a | Describe the results of the search and selection process, from the number of records identified in the search to the number of studies included in the review, ideally using a flow diagram. | 10, Figure 1 |
|  | 16b | Cite studies that might appear to meet the inclusion criteria, but which were excluded, and explain why they were excluded. | Supplementary Section 3 |
| Study characteristics | 17 | Cite each included study and present its characteristics. | 10, Supplementary Section 4 |
| Risk of bias in studies | 18 | Present assessments of risk of bias for each included study. | Supplementary Section 8 |
| Results of individual studies | 19 | For all outcomes, present, for each study: (a) summary statistics for each group (where appropriate) and (b) an effect estimate and its precision (e.g. confidence/credible interval), ideally using structured tables or plots. | 10-14, Table 1,2 |
| Results of syntheses | 20a | For each synthesis, briefly summarise the characteristics and risk of bias among contributing studies. | 14, Supplementary Section 5, 8, 9 |
|  | 20b | Present results of all statistical syntheses conducted. If meta-analysis was done, present for each the summary estimate and its precision (e.g. confidence/credible interval) and measures of statistical heterogeneity. If comparing groups, describe the direction of the effect. | 10-14, Table 1,2 |
|  | 20c | Present results of all investigations of possible causes of heterogeneity among study results. | 10-14, Table 1,2 |
|  | 20d | Present results of all sensitivity analyses conducted to assess the robustness of the synthesized results. | 10-14 |
| Reporting biases | 21 | Present assessments of risk of bias due to missing results (arising from reporting biases) for each synthesis assessed. | 14, Supplementary Section 8 |
| Certainty of evidence | 22 | Present assessments of certainty (or confidence) in the body of evidence for each outcome assessed. | 15, Supplementary Section 9 |
| **DISCUSSION** | | |  |
| Discussion | 23a | Provide a general interpretation of the results in the context of other evidence. | 16-23 |
|  | 23b | Discuss any limitations of the evidence included in the review. | 22-23 |
|  | 23c | Discuss any limitations of the review processes used. | 22-23 |
|  | 23d | Discuss implications of the results for practice, policy, and future research. | 16-23 |
| **OTHER INFORMATION** | | |  |
| Registration and protocol | 24a | Provide registration information for the review, including register name and registration number, or state that the review was not registered. | 5 |
|  | 24b | Indicate where the review protocol can be accessed, or state that a protocol was not prepared. | N/A |
|  | 24c | Describe and explain any amendments to information provided at registration or in the protocol. | N/A |
| Support | 25 | Describe sources of financial or non-financial support for the review, and the role of the funders or sponsors in the review. | 24 |
| Competing interests | 26 | Declare any competing interests of review authors. | 24 |
| Availability of data, code and other materials | 27 | Report which of the following are publicly available and where they can be found: template data collection forms; data extracted from included studies; data used for all analyses; analytic code; any other materials used in the review. | 24 |

*From:*  Page MJ, McKenzie JE, Bossuyt PM, Boutron I, Hoffmann TC, Mulrow CD, et al. The PRISMA 2020 statement: an updated guideline for reporting systematic reviews. BMJ 2021;372:n71. doi: 10.1136/bmj.n71

For more information, visit: <http://www.prisma-statement.org/>

**Section 2: Complete Search Strings**

**Embase: 172 Results**

(**'fallot tetralogy'**/exp OR **'fallot tetralogy'**) AND (**'valve-sparing'** OR **'valve sparing'** OR **'annulus sparing'** OR **'annulus-sparing'**)

**Scopus: 140 Results**

TITLE-ABS-KEY ( ( tetralogy AND of AND fallot ) AND ( ( valve AND sparing ) OR ( valve-sparing ) OR ( annulus AND sparing ) OR ( annulus-sparing ) ) )

**PubMed: 120 Results**

("tetralogy of fallot"[MeSH Terms] OR ("tetralogy"[All Fields] AND "fallot"[All Fields]) OR "tetralogy of fallot"[All Fields]) AND ((("valve"[All Fields] OR "valve s"[All Fields] OR "valved"[All Fields] OR "valves"[All Fields] OR "valving"[All Fields]) AND ("spare"[All Fields] OR "spared"[All Fields] OR "spares"[All Fields] OR "sparing"[All Fields])) OR "valve-sparing"[All Fields] OR ("annulus"[All Fields] AND ("spare"[All Fields] OR "spared"[All Fields] OR "spares"[All Fields] OR "sparing"[All Fields])) OR "annulus-sparing"[All Fields])

**Web of Science: 78 Results**

('fallot tetralogy' OR 'tetralogy of Fallot') AND ('valve-sparing' OR 'valve sparing' OR 'annulus sparing' OR 'annulus-sparing')

**Cochrane Central Register of Controlled Trials (CENTRAL): 1 Result**

('fallot tetralogy' OR 'tetralogy of Fallot') AND ('valve-sparing' OR 'valve sparing' OR 'annulus sparing' OR 'annulus-sparing')

**Section 3: Studies Excluded Due to Overlapping Cohorts**

| **S. No** | **Excluded Study** | **Study Title** | **Country** | **Institute** | **Time Period** | **Overlapping Study** | **Overlapping Outcomes** |
| --- | --- | --- | --- | --- | --- | --- | --- |
| 1 | Balasubramanya et al. (2017) | Right ventricular outflow tract reintervention after primary tetralogy of Fallot repair in neonates and young infants | USA | Boston Children’s Hospital | 2005-2015 | Hofferberth et al. (2017) | - Overall Mortality |
| 2 | Hirji et al. (2010) | Outcomes of prenatally diagnosed tetralogy of Fallot: Implications for valve-sparing repair versus transannular patch. | Canada | The Hospital for Sick Children | 1998-2006 | Hickey et al. (2018) | - Pulmonary Insufficiency - Cardiovascular Reintervention Rate |
| 3 | Lv et al. (2024)* | Feasibility and Surgical Effect of Annulus Sparing in Consecutive Patients with Tetralogy of Fallot: A Retrospective Cohort Study | China | Fuwai Hospital and Cardiovascular Institute | 2014-2017 | Jiang et al. (2020) | - Pulmonary Insufficiency - Cardiovascular Reintervention Rate - Aortic Cross Clamp Time |
| 4 | Robinson et al. (2011)* | The evolving role of intraoperative balloon pulmonary valvuloplasty in valve-sparing repair of tetralogy of Fallot | USA | Boston Children’s Hospital | 1997-2008 | Hofferberth et al. (2017) | - Pulmonary Insufficiency |
| 5 | Stephens et al. (2017)* | Cardiac Function After Tetralogy of Fallot/Complete Atrioventricular Canal Repair, Komansky Center for Children’s Health | USA | New York-Presbyterian Morgan Stanley Children's Hospital | 2005-2014 | Sen et al. (2016) | - Pulmonary Insufficiency - Cardiovascular Reintervention Rate - Residual RVOT Stenosis - Cardiopulmonary Bypass Time - Aortic Cross Clamp Time - Cardiovascular Reintervention Rate - Length of ICU Stay - Length of Hospital Stay |
| *The study was excluded only if data was reported by both studies for a given outcome, and was included in the meta-analysis for other outcomes without overlap. | | | | | | | |

**Section 4: Parametric Estimations of Non-Parametric Data**

*Means and SD’s that have been calculated from Medians, Ranges, and/or IQR’s*

**Postoperative Right Ventricular Outflow Tract Pressure Gradient:**

| **Study** | **Sample Size** | | **TAP** | | **VS** | |
| --- | --- | --- | --- | --- | --- | --- |
|  | **TAP** | **VS** | **Mean** | **SD** | **Mean** | **SD** |
| Sasson et al. (2012) | 20 | 69 | 23.733 | 39.094 | 17.2 | 28.77 |
| Vida et al. (2014) | 32 | 30 | 23.25 | 9.18096 | 23.75 | 7.83162 |
| Stephens et al. (2021) | 17 | 50 | 6.667 | 16.1421 | 17 | 24.4088 |
| Schulte et al. (2023) | 95 | 71 | 23.33 | 8.28 | 23.67 | 10.594 |
| Taksaudom et al. (2024) | 88 | 45 | 22 | 13.784 | 19.167 | 15.074 |

**Postoperative Right Ventricle/Left Ventricle Pressure Ratio**

| **Study** | **Sample Size** | | **TAP** | | **VS** | |
| --- | --- | --- | --- | --- | --- | --- |
|  | **TAP** | **VS** | **Mean** | **SD** | **Mean** | **SD** |
| Sasson et al. (2012) | 20 | 69 | 0.477 | 0.351 | 0.5 | 0.439 |
| Taksaudom et al. (2024) | 88 | 45 | 0.433 | 0.0754 | 0.467 | 0.306 |

**Postoperative Ventilator Time (Hours):**

| **Study** | **Sample Size** | | **TAP** | | **VS** | |
| --- | --- | --- | --- | --- | --- | --- |
|  | **TAP** | **VS** | **Mean** | **SD** | **Mean** | **SD** |
| Minh et al. (2024) | 134 | 398 | 66.57 | 60.553 | 33.83 | 23.139 |
| Taksaudom et al. (2024) | 88 | 45 | 10.33 | 9.798 | 6 | 3.063 |
| Lv et al. (2024)* | 27 | 27 | 37.868 | 38.755 | 18.57 | 12.864 |

**Graphically extracted*

**Postoperative Length of Stay in ICU (Days):**

| **Study** | **Sample Size** | | **TAP** | | **VS** | |
| --- | --- | --- | --- | --- | --- | --- |
|  | **TAP** | **VS** | **Mean** | **SD** | **Mean** | **SD** |
| Vida et al. (2014) | 35 | 34 | 4.75 | 2.6110 | 3.75 | 1.6710 |
| Sen et al. (2016) | 32 | 36 | 4.5 | 1.4496 | 4.5 | 1.4164 |
| Van den Bosch et al. (2014) | 270 | 138 | 3 | 1.4906 | 2.33 | 0.7491 |
| Taksaudom et al. (2024) | 88 | 45 | 1.903 | 1.633 | 0.9304 | 0.1912 |
| Lv et al. (2024)* | 27 | 27 | 3.983 | 1.621 | 2.67 | 1.575 |

**Graphically extracted*

**Postoperative Length of Stay in Hospital (Days):**

| **Study** | **Sample Size** | | **TAP** | | **VS** | |
| --- | --- | --- | --- | --- | --- | --- |
|  | **TAP** | **VS** | **Mean** | **SD** | **Mean** | **SD** |
| Vida et al. (2014) | 35 | 34 | 14.75 | 5.6967 | 17.5 | 8.5939 |
| Sen et al. (2016) | 32 | 36 | 20.25 | 14.2547 | 10 | 4.2493 |
| Schulte et al. (2023) | 95 | 71 | 7 | 3.011 | 6.33 | 2.27 |
| Taksaudom et al. (2024) | 88 | 45 | 8.67 | 4.522 | 6.67 | 3.063 |
| Lv et al. (2024)* | 27 | 27 | 10.826 | 4.654 | 8.562 | 3.163 |

**Graphically extracted*

**Cardiopulmonary Bypass Time (Minutes):**

| **Study** | **Sample Size** | | **TAP** | | **VS** | |
| --- | --- | --- | --- | --- | --- | --- |
|  | **TAP** | **VS** | **Mean** | **SD** | **Mean** | **SD** |
| Vida et al. (2014) | 35 | 34 | 164.25 | 31.5690 | 104.5 | 25.5431 |
| Sen et al. (2016) | 32 | 36 | 125 | 43.0056 | 108.75 | 21.9547 |
| Hickey et al. (2018) | 138 | 196 | 150.75 | 51.9062 | 130.5 | 35.1889 |
| Ono et al. (2022) | 242 | 198 | 183.33 | 57.43 | 155.33 | 34.35 |
| Schulte et al. (2023) | 95 | 71 | 106.67 | 24.09 | 99 | 27.2714 |
| Taksaudom et al. (2024) | 88 | 45 | 110.83 | 45.22 | 89.33 | 45.95 |

**Aortic Cross-Clamp Time (Minutes):**

| **Study** | **Sample Size** | | **TAP** | | **VS** | |
| --- | --- | --- | --- | --- | --- | --- |
|  | **TAP** | **VS** | **Mean** | **SD** | **Mean** | **SD** |
| Vida et al. (2014) | 35 | 34 | 78 | 15.6658 | 71 | 24.8269 |
| Sen et al. (2016) | 32 | 36 | 68.5 | 19.3283 | 72 | 11.8036 |
| Hickey et al. (2018) | 138 | 196 | 92 | 27.9642 | 73.75 | 22.9095 |
| Van den Bosch et al. (2019) | 188 | 110 | 78.667 | 35.8476 | 85.333 | 41.2913 |
| Ono et al. (2022) | 242 | 198 | 91.33 | 23.865 | 85.67 | 19.416 |
| Schulte et al. (2023) | 95 | 71 | 64.33 | 17.314 | 63 | 21.944 |
| Taksaudom et al. (2024) | 88 | 45 | 85.67 | 33.163 | 66.33 | 43.65 |

**Section 5: Study and Participant Characteristics**

| **Table A: Study Characteristics**  *Robinson et al. described two valve-sparing approaches: commissurotomy or standard rigid dilation (Hegar), and sequential intraoperative balloon pulmonary valvuloplasty (IBPV).  *Abbreviations: PSM: Propensity Score Matching; IQR: Interquartile Range; USA: United States of America; QCHDD: Quebec Congenital Heart Disease Database; PCCC: Pediatric Cardiac Care Consortium; NDI: National Death Index; OPTN: Organ Procurement and Transplantation Network.* | | | | | | | | | | | |
| --- | --- | --- | --- | --- | --- | --- | --- | --- | --- | --- | --- |
| **S. No** | **Study** | **Year** | **Country** | **Study Design** | **PSM** | **Time Period** | **Centers** | **Institution(s)** | **Data Source** | **ROBINS-I Risk of Bias** | **Average Follow-Up Time** |
| 1 | **Annavajjhala et al.** | 2019 | USA | Retrospective Cohort | No | 2002-2015 | 1 | Lucile Packard Children’s Hospital Stanford | Institutional | Moderate | - |
| 2 | **Arslan et al.** | 2013 | Türkiye | Retrospective Cohort | No | 2006-2009 | 1 | Kartal Kosuyolu Research and Training Hospital | Institutional | Moderate | - |
| 3 | **Aydin et al.** | 2018 | Türkiye | Retrospective Cohort | No | 2010-2015 | 1 | Acibadem University Hospital Atakent | Institutional | Moderate | Median (Range): 6.5 (1 - 24) months |
| 4 | **Blais et al.** | 2021 | Canada | Retrospective Cohort | Yes | 1980-2015 | - | - | QCHDD | Low | Median (IQR): 16.0 (8.1 - 25.4) years |
| 5 | **Bonura et al.** | 2023 | USA | Retrospective Cohort | No | 2006-2016 | 1 | Saint Louis University School of Medicine | Institutional | Serious | Median (IQR): 7.3 (4.7–9.1) years |
| 6 | **Borodinova et al.** | 2019 | Ukraine | Prospective Cohort | No | 2016-2017 | 1 | Ukrainian Children Cardiac Center | Institutional | Low | - |
| 7 | **Bové et al.** | 2011 | Belgium | Retrospective Cohort | No | 1994-2010 | 1 | University Hospital of Gent | Institutional | Moderate | Median (Range): 7.7 years (6 months - 16 years) |
| 8 | **Escribano et al.** | 2011 | Spain | Retrospective Cohort | No | 2001-2009 | 1 | Hospital Universitario 12 de Octubre | Institutional | Serious | Mean ± SD: 42.5 ± 29.7 months |
| 9 | **Gupta et al.** | 2012 | USA | Retrospective Cohort | No | 1988-2008 | 1 | Rush Center for Congenital and Structural Heart Disease | Institutional | Moderate | Median (IQR): 9.2 (4.7 - 13.3) years |
| 10 | **Hickey et al.** | 2018 | Canada | Retrospective Cohort | No | 2000-2012 | 1 | The Hospital for Sick Children | Institutional | Moderate | Median: 5.6 years |
| 11 | **Hiroki Ito et al.** | 2013 | Japan | Retrospective Cohort | No | 2005-2011 | 1 | Mt. Fuji Shizuoka Children’s Hospital | Institutional | Serious | Mean ± SD: 2.6 ± 2.4 years |
| 12 | **Hofferberth et al.** | 2017 | USA | Retrospective Cohort | Yes | 2007-2015 | 1 | Boston Children's Hospital | Institutional | Low | Median (IQR): 2.5 (0.6 - 4.9) years |
| 13 | **Hua et al.** | 2011 | China | Retrospective Cohort | No | 2006-2010 | 1 | Fuwai Hospital and Cardiovascular Institute | Institutional | Serious | 27 months |
| 14 | **Ismail et al.** | 2010 | Saudi Arabia | Retrospective Cohort | No | 2002-2007 | 1 | King Abdulaziz Cardiac Center | Institutional | Moderate | - |
| 15 | **Jiang et al.** | 2020 | China | Retrospective Cohort | Yes | 2012-2017 | 1 | Fuwai Hospital and Cardiovascular Institute | Institutional | Low | Median (Range): TAP: 50 (6–90); VS: 48 (9–93) months |
| 16 | **Kim et al.** | 2014 | South Korea | Retrospective Cohort | Yes | 1989-2005 | 1 | Asan Medical Center | Institutional | Low | 146 months |
| 17 | **Kobayashi et al.** | 2022 | Japan | Retrospective Cohort | No | 1991-2019 | 1 | Okayama University Hospital | Institutional | Moderate | Mean ± SD: 15 ± 7.3 years |
| 18 | **Lv et al.** | 2022 | China | Retrospective Cohort | Yes | 2014-2017 | 1 | Fuwai Hospital and Cardiovascular Institute | Institutional | Low | Mean ± SD: (30.3 ± 11.6) |
| 19 | **Mahajan et al.** | 2019 | USA | Retrospective Cohort | No | 2000-2017 | 1 | Riley Hospital for Children | Institutional | Serious | Median: 7.6 years |
| 20 | **Mandigma et al.** | 2016 | Philippines | Prospective Cohort | No | 2012-2014 | 1 | Philippine Heart Center | Institutional | Low | - |
| 21 | **Minh et al.** | 2024 | Vietnam | Retrospective Cohort | No | 2010-2020 | 1 | Vietnam National Children’s Hospital | Institutional | Moderate | Median 4.6 years (interquartile range, 2.9–6.1 years). |
| 22 | **Mouws et al.** | 2018 | Netherlands | Retrospective Cohort | No | 2000-2015 | 1 | Erasmus Medical Center | Institutional | Moderate | - |
| 23 | **Ono et al.** | 2022 | Japan | Retrospective Cohort | Yes | 1978-2003 | 1 | National Cerebral and Cardiovascular Center | Institutional | Low | Median 20.3 years (interquartile range, 10.7-  27.6). |
| 24 | **Padalino et al.** | 2017 | Italy | Retrospective Cohort | No | 1990-2004 | 3 | Padova University Hospital, Bambino Gesù Children's Hospital, Ospedale Regina Margherita | Institutional | Moderate | Median (IQR): 16.6 (12.5-20.3) years |
| 25 | **Pawan et al.** | 2020 | India | Retrospective Cohort | No | 2019 | 1 | G. B. Pant Hospital | Institutional | Serious | - |
| 26 | **Robinson et al.** | 2011 | USA | Retrospective Cohort | No | 1997-2008 | 1 | Boston Children's Hospital | Institutional | Serious | Median (Range): TAP: 5.1 (0–11.6) years; IBPV: 1.5 (0.4–3.5) years; Hegar: 5.8 (0–11.9) years* |
| 27 | **Sasson et al.** | 2012 | Israel | Retrospective Cohort | No | 2003-2009 | 1 | Wolfson Medical Center | Institutional | Serious | Mean: Trivial PI: 26.4 months; Mild PI: 30.8 months; Moderate PI: 23.2 months; Free PI: 26.3 months |
| 28 | **Schulte et al.** | 2023 | USA | Retrospective Cohort | No | 2007-2021 | 1 | Washington University School of Medicine | Institutional | Serious | 44 months |
| 29 | **Sen et al.** | 2016 | USA | Retrospective Cohort | No | 2010-2014 | 1 | New York-Presbyterian Morgan Stanley Children's Hospital | Institutional | Low | Median (Range): 9 (1–59) months |
| 30 | **Simon et al.** | 2017 | USA | Retrospective Cohort | No | 2000-2010 | 2 | University of Rochester Medical Center, SUNY Upstate Medical University | Institutional | Serious | Mean ± SD: 7.9 ± 3.4 years. |
| 31 | **Singab et al.** | 2020 | Egypt | Retrospective Cohort | No | 2012-2019 | 1 | Ain Shams University Specialized Hospital | Institutional | Serious | 6 years |
| 32 | **Singh et al.** | 2011 | India | Prospective Cohort | No | 2008 | 1 | G. B. Pant Hospital | Institutional | Low | - |
| 33 | **Smith et al.** | 2018 | USA | Retrospective Cohort | No | 1982-1989; 1990-1999; 2000-2006 | - | - | PCCC, NDI, OPTN | Moderate | Median (IQR): 18.5 (14.6-22.4) years |
| 34 | **Stephens et al.** | 2021 | USA | Retrospective Cohort | No | 2008-2018 | 1 | Ann & Robert H. Lurie Children’s Hospital of Chicago | Institutional | Serious | Median (IQR): 42 (16-73) months |
| 35 | **Stephens et al.** | 2017 | USA | Retrospective Cohort | No | 2005-2014 | 2 | New York-Presbyterian Morgan Stanley Children's Hospital, Komansky Center for Children’s Health | Institutional | Serious | Mean ± SD: 3.0 ± 3.0 |
| 36 | **Stewart et al.** | 2005 | USA | Retrospective Cohort | No | 1997-2004 | 1 | Ann & Robert H. Lurie Children's Hospital of Chicago | Institutional | Serious | Mean (Range): 34 (4 – 84) months |
| 37 | **Taksaudom et al.** | 2024 | Thailand | Retrospective Cohort | Yes | 2006-2016 | 1 | Chiang Mai University | Institutional | Low | 79.8 months (interquartile range: 50.7-115.5 months. |
| 38 | **van den Bosch et al.** | 2020 | Netherlands | Retrospective Cohort | No | 1970-2012 | 1 | Erasmus Medical Centre, Radboud Medical Centre | Institutional | Moderate | Median (IQR): 16.2 (9.2–22.8) years |
| 39 | **Vida et al.** | 2014 | Italy | Retrospective Cohort | No | 2007-Unspecified | 1 | Padova University Hospital | Institutional | Serious | Median (Range): 580 (189-1940) days |
| 40 | **Wu et al.** | 2021 | China | Retrospective Cohort | No | 2009-2019 | 3 | The Second Xiangya Hospital of Central South University, Hunan Children’s Hospital, Hunan Provincial People’s Hospital | Institutional | Low | Range: 3 - 12 years |

| **Table B: Preoperative Participant Characteristics**  *****Value not given, but the comparison was reported as insignificant, or the variable was included in the propensity score matching model.  ^+^Value not given but were reported to be significantly different, or based on study inclusion criteria the groups were determined to be significantly different from each other.  ^‡^ Escribano et al. did not report immediate preoperative PVA Z-scores, the values reported are the ultrasound findings at last fetal diagnostic echocardiography.  ^α^ Mahajan et al. data in the table corresponds to data reported at “complete repair”, not at the initial shunt placement.  ^β^ Robinson et al. described two valve-sparing approaches: commissurotomy or standard rigid dilation (Hegar), and sequential intraoperative balloon pulmonary valvuloplasty (IBPV). For the purposes of meta-analysis, both approaches were combined to form the VS group that was pooled for postoperative outcomes given there were no significant differences in postoperative outcomes between the Hegar and IBPV groups.  *Abbreviations: VS: Valve-Sparing; TAP: Transannular Patch; IQR: Interquartile Range; PVA: Pulmonary Valve Annulus; SD: Standard Deviation* | | | | | | | | | | | | | | |
| --- | --- | --- | --- | --- | --- | --- | --- | --- | --- | --- | --- | --- | --- | --- |
| **S. No** | **Study** | **Year** | **Study Participants** | | | **Age** | | **P-value** | **Weight (kg)** | | **P-value** | **PVA Z-Score** | | **P-value** |
|  |  |  | **VS** | **TAP** | **Total** | **VS** | **TAP** |  | **VS** | **TAP** |  | **VS** | **TAP** |  |
| 1 | **Annavajjhala et al.** | 2019 | 18 | 20 | 38 | 72.2 ± 8.6 months | 70.4 ± 12.7 months | -* | - | - | - | - | - | - |
| 2 | **Arslan et al.** | 2013 | 21 | 127 | 148 | 3.6 ± 3.5 years | 4.7 ± 2.9 years | 0.12 | 17.4 ± 10.7 | 13.5 ± 6.3 | **0.02** | - | - | -+ |
| 3 | **Aydin et al.** | 2018 | 29 | 35 | 64 | 22.8 ± 16.9 months | 17.5 ± 11.0 months | **0.035** | 10.7 ± 3.47 | 9.4 ± 2.2 | 0.129 | - | - | - |
| 4 | **Blais et al.** | 2021 | 264 | 264 | 528 | Median (IQR): 0.97 (0.51 - 1.86) years | Median (IQR): 0.85 (0.38 - 2.00) years | **0.01** | - | - | - | - | - | -* |
| 5 | **Bonura et al.** | 2023 | 43 | 19 | 62 | - | - | - | 6.73 ± 1.24 | 6.42 ± 1.14 | 0.36 | -1.30 ± 1.08 | -2.56 ± 0.71 | **<0.001** |
| 6 | **Borodinova et al.** | 2019 | 42 | 16 | 58 | Median (Range) 7.6 (2.1 - 46.3) months | Median (Range) 7.0 (0.9 - 41.7) months | 0.37 | Median (Range): 7.8 (4.2 - 15.5) | Median (Range): 6.7 (3.2 - 24.4) | 0.14 | -3.0 ± 1.9 | -3.9 ± - 2 | 0.063 |
| 7 | **Bové et al.** | 2011 | 48 | 47 | 95 | Median: 7 months | Median: 7.5 months | 0.21 | - | - | - | −0.86 ± 1.15 | −3.26 ± 0.95 | **<0.001** |
| 8 | **Escribano et al.** | 2011 | 11 | 11 | 22 | Mean (SD, range): 20.9 (0.9, 19 - 22) weeks | | - | Mean (SD, Range): 2.867 (0.594, 1.940 – 4.150) | | - | -2.3 ± 1.1‡ | -3.3 ± 1.1‡ | **0.041** |
| 9 | **Gupta et al.** | 2012 | 8 | 5 | 13 | 6.3 ± 2.4 months | | - | - | - | - | - | - | -+ |
| 10 | **Hickey et al.** | 2018 | 296 | 138 | 434 | Median (Range): 182.5 (29 - 5527) days | Median (Range): 171.5 (16 - 4235) days | 0.576 | Median (Range): 6.9 (2.1–67) | Median (Range): 6.8 (2.5–85) kg | 0.718 | Median: -4.5 | Median: -6.9 | **<0.001** |
| 11 | **Hiroki Ito et al.** | 2013 | 11 | 8 | 19 | Median (IQR): 6.9 (2.2-16.1) months | Median (IQR): 8.7 (1.0- 13) months | 0.65 | Median (IQR): 6.9 (4.6 to 9.2) | Median (IQR): 6.4 (3.6 to 8.9) | 0.68 | Median (IQR): −4.9 (−6.3 to −4.3) | Median (IQR): −6.1 (−7.9 to −4.3) | **0.04** |
| 12 | **Hofferberth et al.** | 2017 | 53 | 53 | 106 | 96 ± 55 days | 83 ± 49 days | 0.181 | - | - | - | -2.21 ± 0.55 | -2.26 ± 0.65 | 0.67 |
| 13 | **Hua et al.** | 2011 | 132 | 7 | 139 | 8.1 ± 3.2 months | 7.5 ± 2.1 months | - | 7.8 ± 6.2 | 7.5 ± 3.7 | - | 1.5 ± 1.1 | -4.7 ± 1.2 | **0.001** |
| 14 | **Ismail et al.** | 2010 | 19 | 64 | 83 | 15.6 ± 16 months | 18.8 ± 24.4 months | 0.06 | 6.9 ± 3.4 | 8.6 ± 4.4 | 0.12 | - | - | -+ |
| 15 | **Jiang et al.** | 2020 | 944 | 729 | 1673 | Median (Range) 320 (54–6497) days | Median (Range): 318 (57–5969) days | 0.934 | 10.1 ± 5.5 | 10.0 ± 5.0 | 0.829 | Median (IQR): -2.27 (-4.24-2.16) | Median (IQR): 2.34 (-4.26-2.52) | 0.51 |
| 16 | **Kim et al.** | 2014 | 57 | 57 | 114 | 9.3 ± 1.9 months | 9.3 ± 2.2 months | 0.891 | 8.2 ± 1.3 | 8.3 ± 1.3 | 0.702 | -2.3 ± 1.3 | -2.1 ± 1.3 | 0.547 |
| 17 | **Kobayashi et al.** | 2022 | 207 | 123 | 330 | Median (IQR): 428 (317-570) days | Median (IQR): 527 (331-714) days | 0.081 | Median (IQR): 8.4 (7.4-9.6) | Median (IQR): 9 (7.9-10.3) | **0.018** | -1.5 ± 1.7 | -3.5 ± 1.5 | **<0.001** |
| 18 | **Lv et al.** | 2022 | 27 | 27 | 290 | Median (IQR): 13.0 (6.6-24.0) months | Median (IQR): 11.0 (8.0-19.0) months | 0.945 | 11.6 ± 9.1 | 11.9 ± 9.6 | 0.885 | −3.2 ± 1.9 | −3.5 ± 0.9 | 0.208 |
| 19 | **Mahajan et al.** ^α^ | 2019 | 25 | 34 | 59 | Median (IQR): 12.3 (10.4-14.0) months | Median (IQR): 11.3 (9.7-15.3) months | 0.69 | 7.8 ± 1.5 | 8.3 ± 1.4 | 0.69 | - | - | - |
| 20 | **Mandigma et al.** | 2016 | 40 | 23 | 63 | 7.0 + 3.8 years | 7.5 + 3.5 years | 0.64 | 19.5 + 9.89 | 22.5 + 10.64 | 0.27 | - 2.1 ± 1.1 | - 2.5 ± 1.7 | 0.27 |
| 21 | **Minh et al.** | 2024 | 398 | 134 | 532 | Median (IQR): 12.0 (8.9–18.3) months | Median (IQR): 11.7 (8.7–16.9) months | 0.66 | - | - | - | Median (IQR): −0.68 (−1.41-0.03) | Median (IQR): −2.64 (−3.69 to -2.02) | **<0.001** |
| 22 | **Mouws et al.** | 2018 | 57 | 120 | 177 | Median (IQR): 3.5 (2.6 - 5.2) months | | - | Mean ± SD (Range) 5.8 ± 2.2 (2 - 15.7) | | - | - | - | -+ |
| 23 | **Ono et al.** | 2022 | 198 | 242 | 440 | Median (IQR): 1.6 (0.9-2.5) years | Median (IQR): 1.9 (1.3-2.7) years | **0.001** | Median (IQR): 10.4 (8.7-12.1) | Median (IQR): 9.3 (7.9-11.7) | **0.002** | Median (IQR): 0.05 (-0.39-0.66) | Median (IQR): -1.89 (-3.11 to -0.95) | **<0.001** |
| 24 | **Padalino et al.** | 2017 | 18 | 61 | 79 | Median (IQR): 1.28 (0.63-1.95) years | Median (IQR): 0.92 (0.7-1.13) years | 0.149 | - | - | - | - | - | -+ |
| 25 | **Pawan et al.** | 2020 | 10 | 19 | 29 | 18.52 ± 3.45 years | 17.28 ± 3.22 years | 0.35 | 35 ± 6.8 | 36 ± 5.9 | 0.99 | -1.2 ± 0.31 | -2.75 ± 0.26 | **0.001** |
| 26 | **Robinson et al.** ^β^ | 2011 | 127 | 111 | 238 | IBPV: 94 ± 35 days; Hegar: 93 ± 41 days | 74 ± 45 days | -+ (for Hegar) | IBPV: 5.21 ± 0.89; Hegar 5.04 ± 1.35 | 4.56 ± 1.46 | -* | IBPV -2.40 ± 0.96; Hegar -1.43 ± 1.09 | -2.79 ± 0.95 | -+ (for Hegar); -* (for IBPV |
| 27 | **Sasson et al.** | 2012 | 69 | 20 | 163 | Median (Range): 27 (4–192) months | Median (Range): 36 (3–226) months | 0.247 | Median (Range): 11.7 (4.3–49) | Median (Range): 9.35 (2.8–42.7) | 0.396 | Median (Range): 0 (−2 to 1) | Median (Range): −3.1 (−5 to 0.5) | **<0.0001** |
| 28 | **Schulte et al.** | 2023 | 71 | 95 | 166 | Median (IQR): 4.8 (3.5-6.6) months | Median (IQR): 5 (3.8-6.0) months | 0.39 | Median (IQR): 6 (5.3-7.0) | Median (IQR): 6.0 (4.98-7.3) | 0.49 | Median (IQR): -2.0 (-2.8 to -0.8) | Median (IQR): -3.7 (-4.4 to -3.1) | **<0.001** |
| 29 | **Sen et al.** | 2016 | 19 | 32 | 80 | Median (Range): 109 (5–329) days | Median (Range): 156 (6–350) days | 0.279 | Median (Range): 6.0 (1.9–8.5) | Median (Range): 5.6 (2.3–10.5) | 0.941 | Median (Range): -2.51 (-5.80 to 0.49) | Median (Range): -3.07 (-9.00 to 0.80) | 0.065 |
| 30 | **Simon et al.** | 2017 | 42 | 48 | 92 | 101.9 ± 60.9 days | 147.7 ± 83.2 days | 0.002 | 6.1 ± 1.7 | 5.6 ± 1.8 | 0.17 | –0.91 ± 1.56 | –2.72 ± 1.35 | **<0.001** |
| 31 | **Singab et al.** | 2020 | 103 | 377 | 480 | 19 ± 8 months | 18 ± 9 months | 0.06 | 7.8 ± 3.4 | 8.8 ± 4.3 | 0.12 | - | - | -+ |
| 32 | **Singh et al.** | 2011 | 20 | 23 | 43 | Mean (Range): 28 (6–260) months | Mean (Range): 26 (6–180) months | 0.19 | Mean ± SD (Range): 10.1 ± 5.9 (6–25) | Mean ± SD (Range): 10.6 ± 4.8 (6–54) | 0.76 | −2.8 ± 1.5 | −3.0 ± 1.2 | 0.64 |
| 33 | **Smith et al.** | 2018 | 1179 | 2549 | 3894 | - | - | - | - | - | - | - | - | - |
| 34 | **Stephens et al.** | 2021 | 50 | 17 | 67 | Median (IQR): 4.9 (2.9 to 5.7) months | Median (IQR): 4.5 (3.5 to 6.0) months | 0.23 | 4.7 ± 0.9 | 8.0 ± 2.1 | **<0.001** | 0.87 ± 0.89 | -1.1 ± 1.1 | **<0.001** |
| 35 | **Stephens et al.** | 2017 | 6 | 14 | 20 | 94 ± 142 weeks | 21 ± 10 weeks | 0.494 | Median (IQR): 5.7 (5.1 to 6.7) | Median (IQR): 6.4 (5.3 to 7.0) | 0.559 | Median (IQR): -2.0 (2.7 to 0.7) | Median (IQR): -3.1 (4.1 to 2.9) | **<0.001** |
| 36 | **Stewart et al.** | 2005 | 82 | 20 | 102 | 11 ± 8.4 months | 9.4 ± 19 months | 0.7 | 7.4 ± 5.8 | 7.5 ± 2.8 | 0.9 | -1.7 ± 1.2 | -4.8 ± 1.7 | **<0.001** |
| 37 | **Taksaudom et al.** | 2024 | 45 | 88 | 133 | Median (IQR): 40 (27-56.5) months | Median (IQR): 41 (33-55) months | 0.708 | Median (IQR): 12 (11-14.5) | Median (IQR): 12.5 (11.2-14.5) | 0.149 | Median (IQR): -0.8 (-2.1 to 0.1) | Median (IQR): -2.3 (-3.6 to -1.2) | **<0.001** |
| 38 | **van den Bosch et al.** | 2020 | 159 | 294 | 453 | Median (IQR): 0.7 (0.3–1.3) years | Median (IQR): 0.7 (0.4–1.4) years | 0.15 | Median (IQR): 7.3 (5.3–9.5) | Median (IQR): 7.1 (5.3–8.9) | 0.42 | - | - | - |
| 39 | **Vida et al.** | 2014 | 34 | 35 | 69 | Median (Range): 115 (36-521) days | Median (Range): 113 (65-454) days | 0.41 | Median (Range): 5.7 (4.6-10) | Median (Range): 6.0 (3.2-10.1) | 0.92 | Median (Range): -2.95 (-0.95 to -4.06) | Median (Range): -3.35 (-1.54 to -5.62) | **0.03** |
| 40 | **Wu et al.** | 2021 | 63 | 65 | 128 | 12.6 ± 4.8 months | 14.2 ± 6.7 months | 0.113 | 9.32 ± 1.63 | 9.12 ± 1.16 | 0.423 | −2.58 ± 0.30 | −2.61 ± 0.26 | 0.502 |

**Section 6: Forest Plots and Additional Results**

**Legend:** SD, standard deviation; RR, risk ratio; MD, mean difference; M-H, Mantel-Haenszel; IV, inverse variance; CI, confidence interval; df, degrees of freedom; P, probability value

***Subsection 1: Intraoperative Outcomes***

**Supplementary Figure 6.1.1: Aortic Cross-Clamp Time (Minutes)**

**
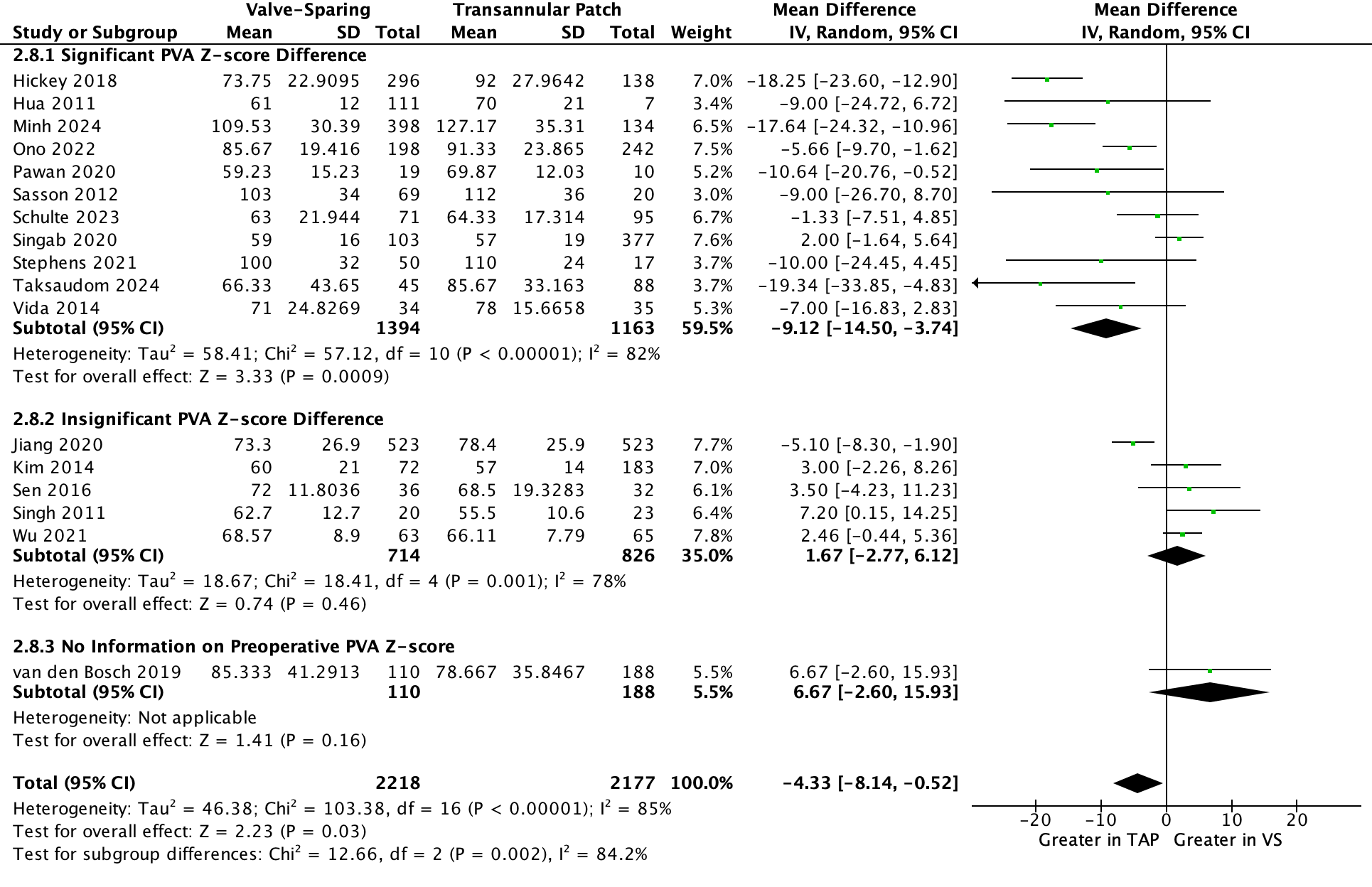
**

**Supplementary Figure 6.1.2: Cardiopulmonary Bypass Time (Minutes)**

***
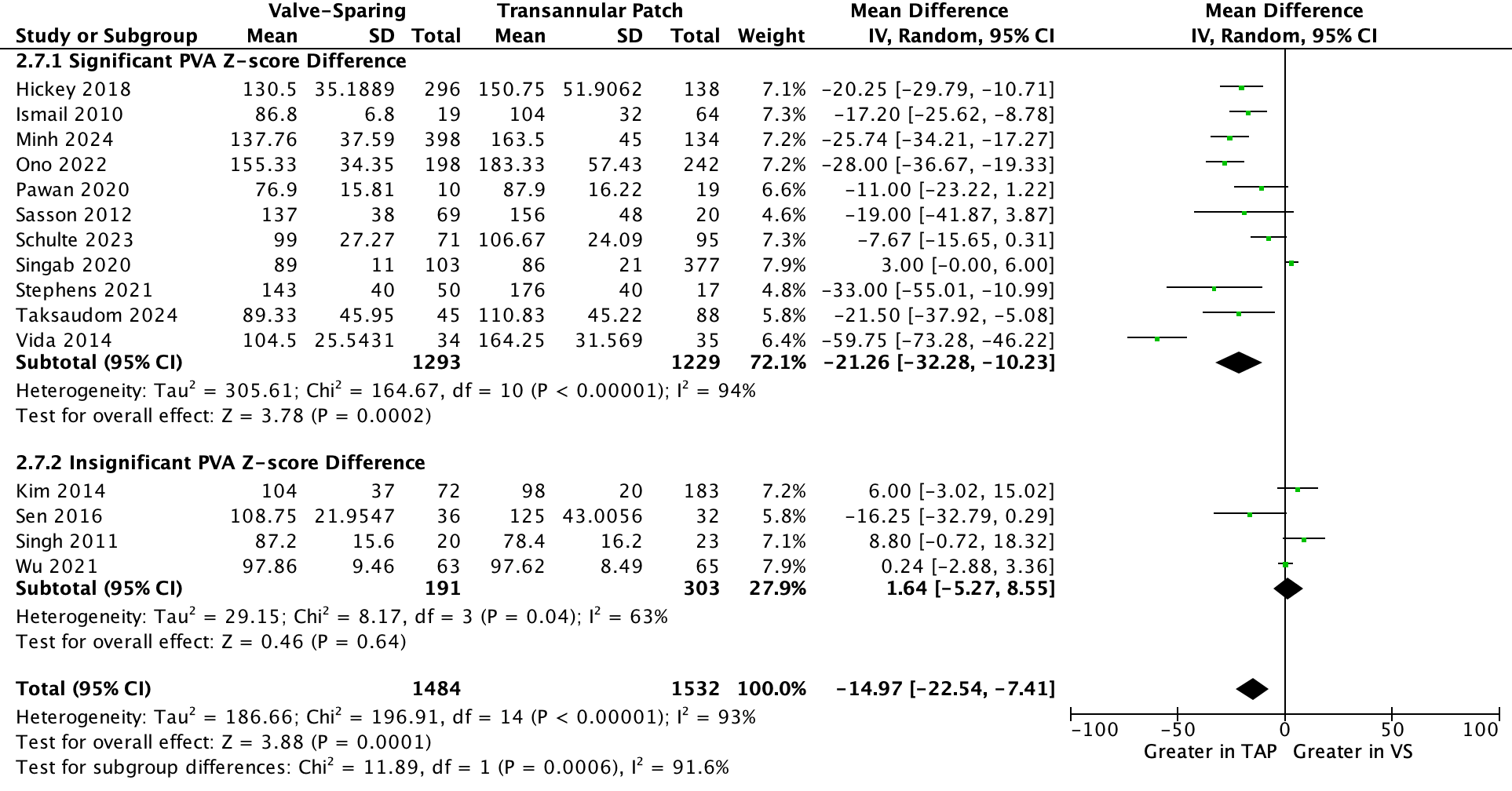
***

***Subsection 2: Postoperative Clinical Outcomes***

**Supplementary Figure 6.2.1: Cardiovascular Reintervention Rate**

**
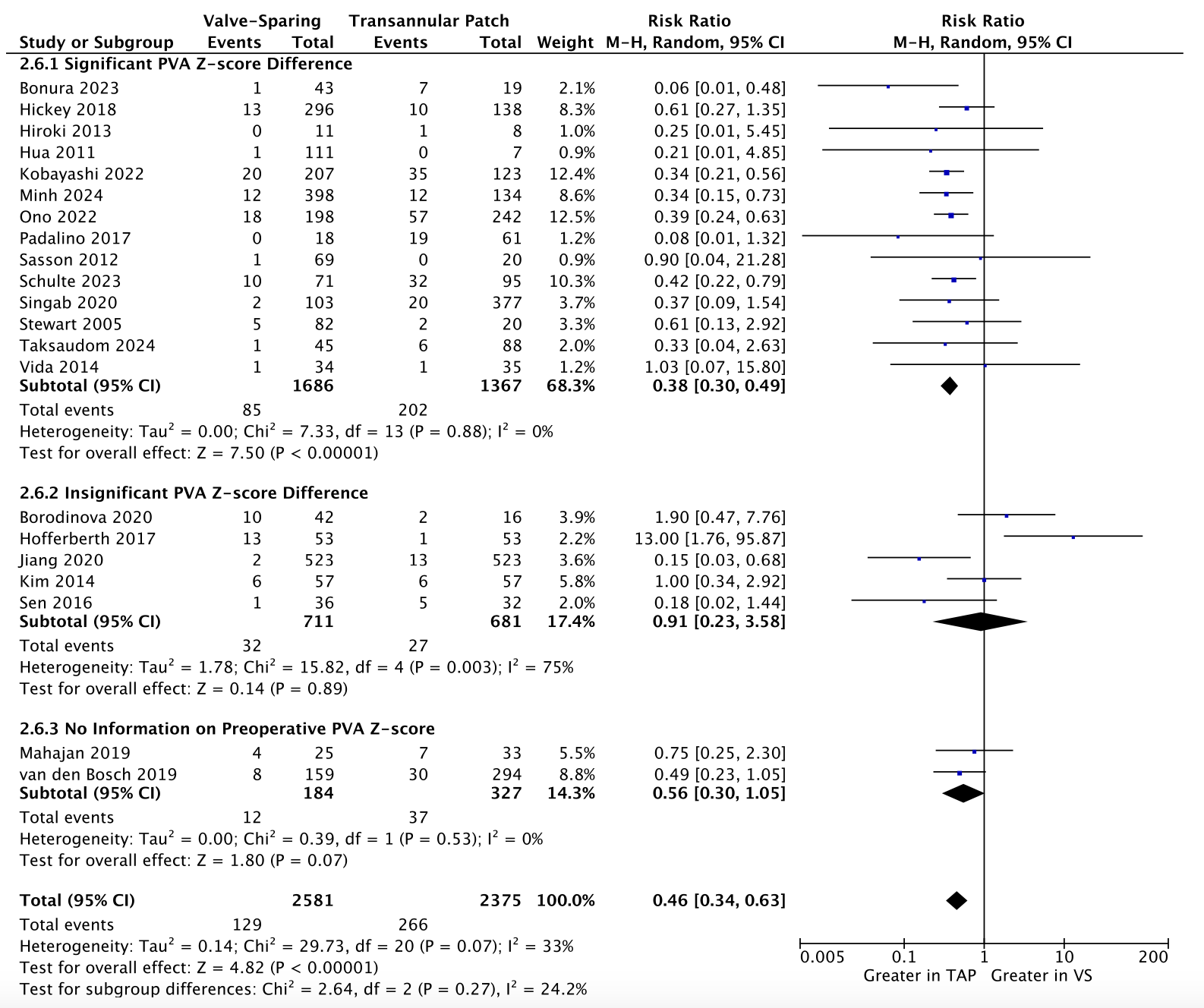
**

**Supplementary Figure 6.2.2: Overall Mortality**

**
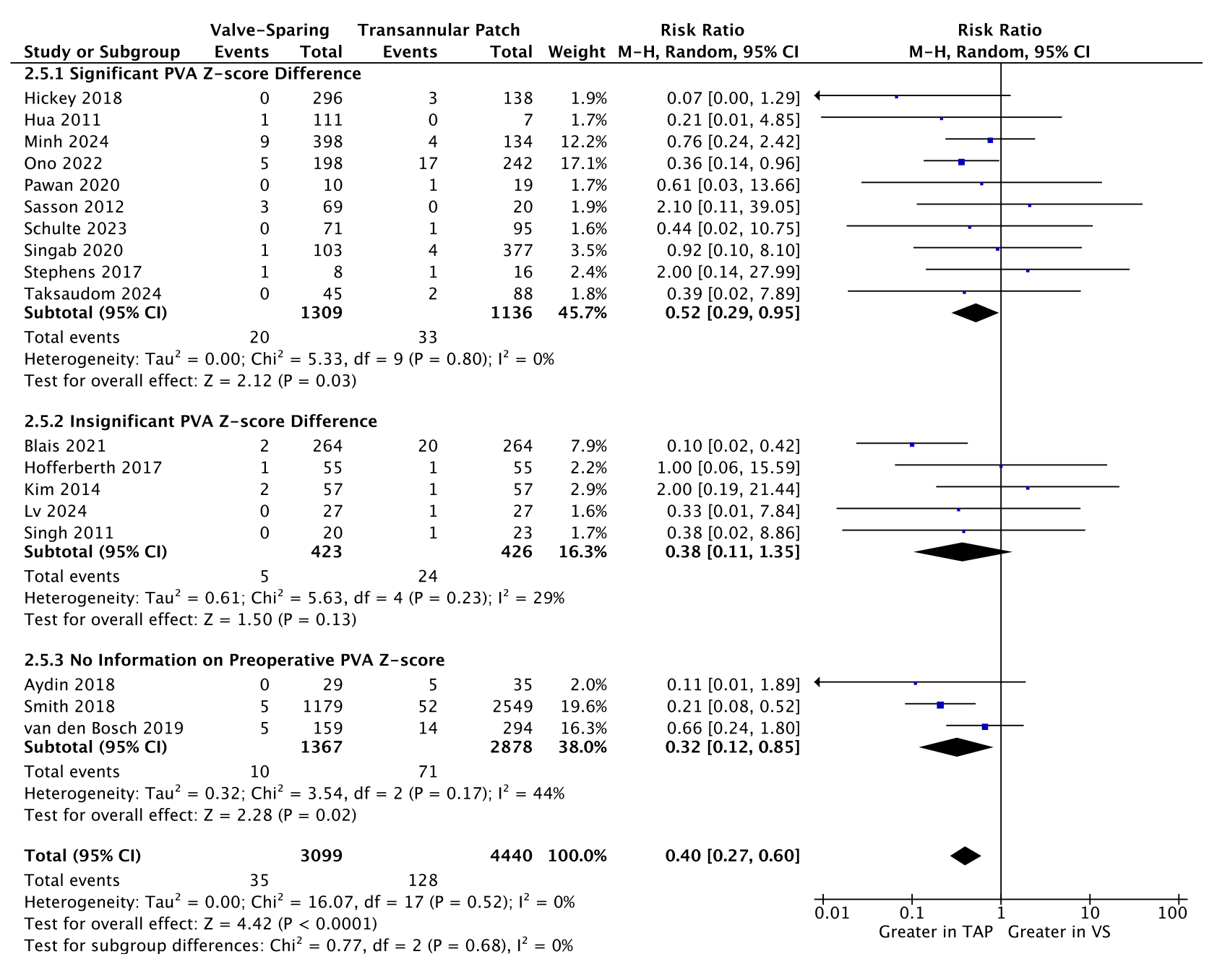
**

**Supplementary Figure 6.2.3: Length of ICU Stay (Days)**

**
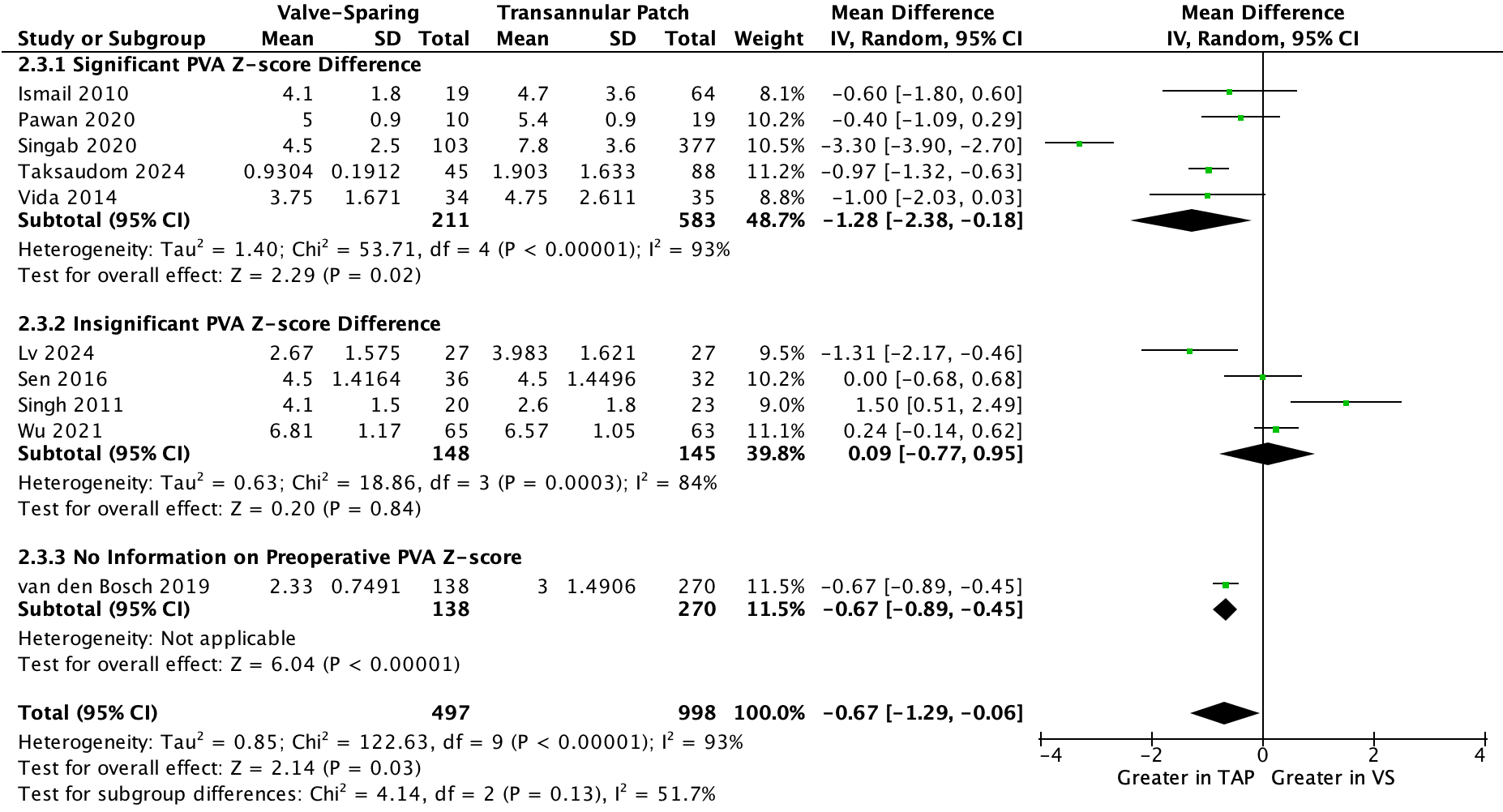
**

**Supplementary Figure 6.2.4: Ventilation Duration (Hours)**

**
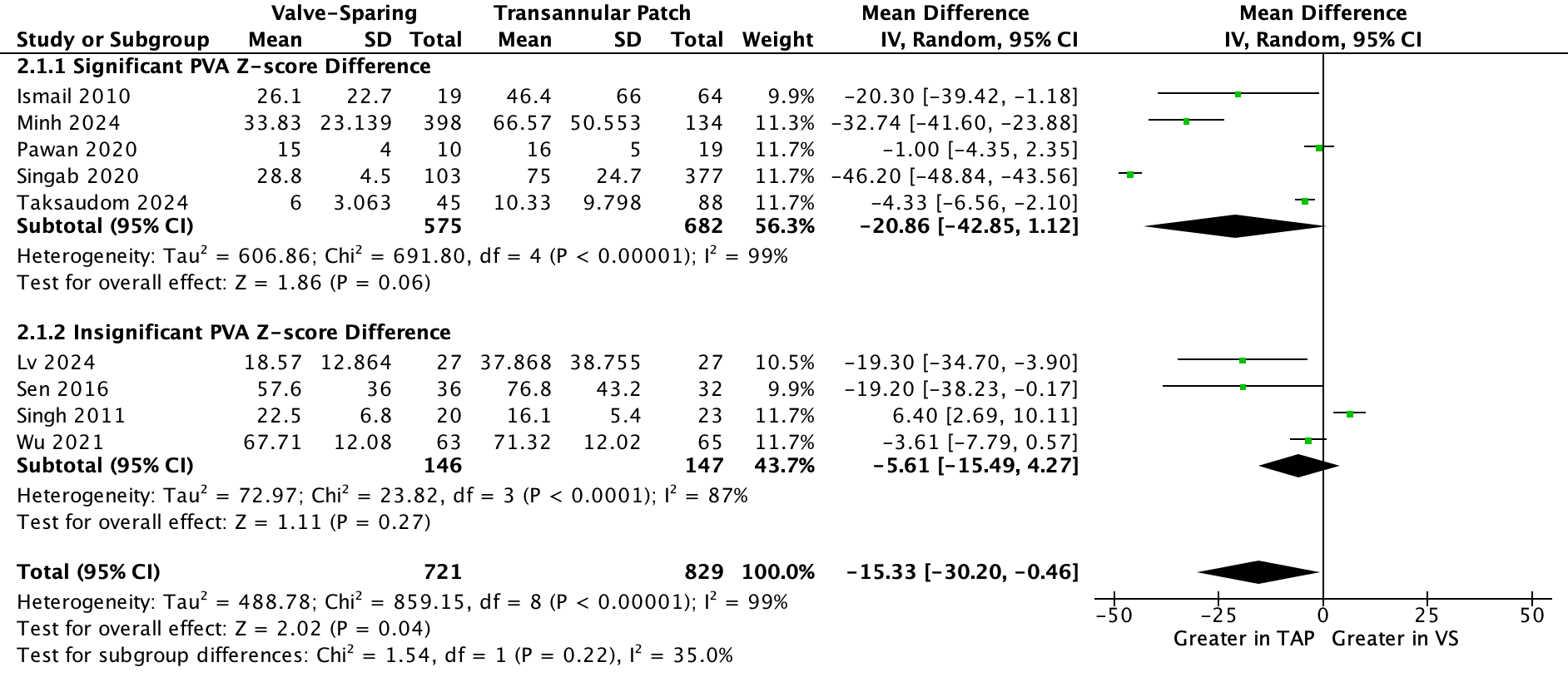
**

**Supplementary Figure 6.2.5: Arrhythmia**

**
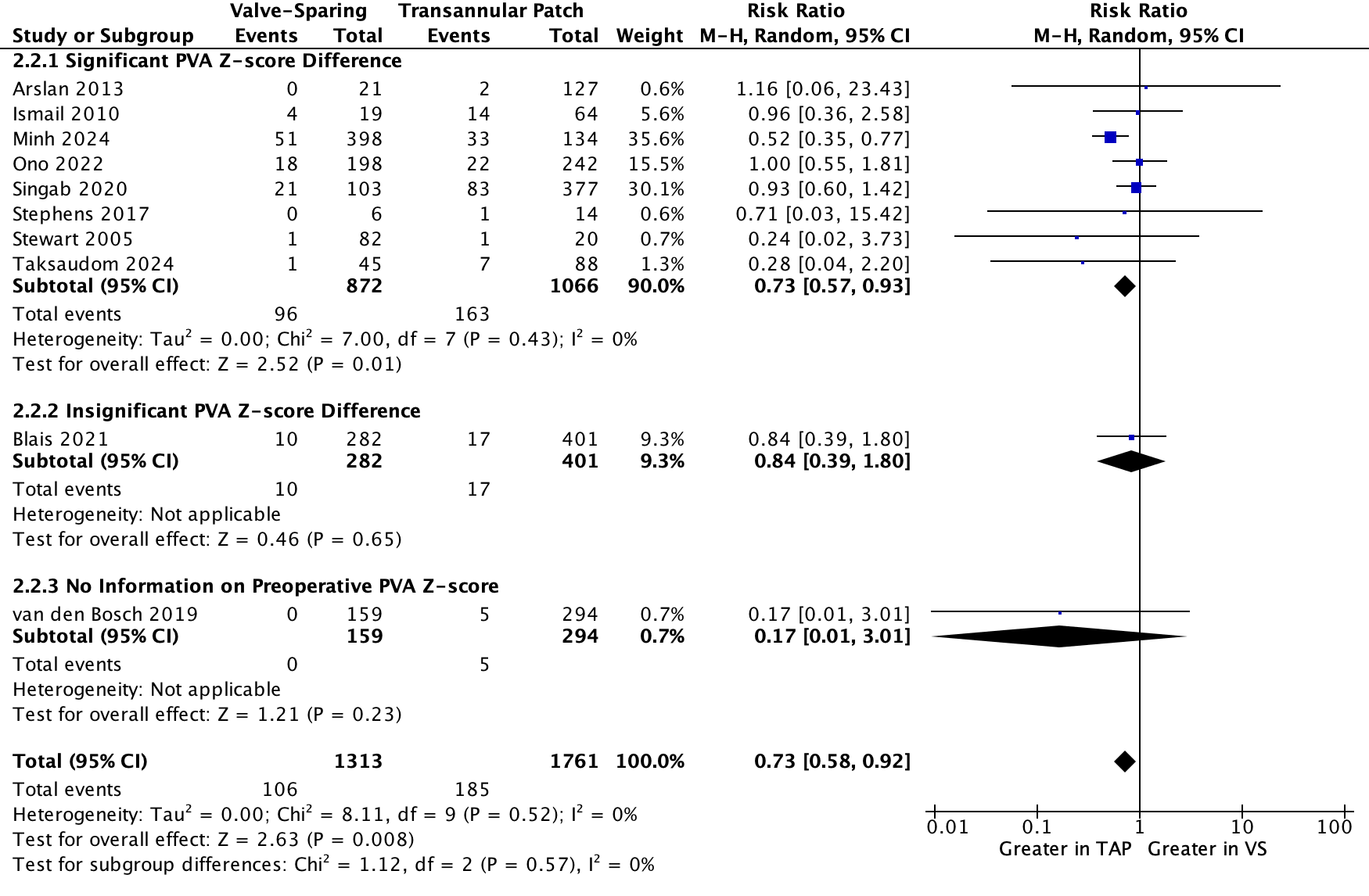
**

**Supplementary Figure 6.2.6: Length of Hospital Stay (Days)**

**
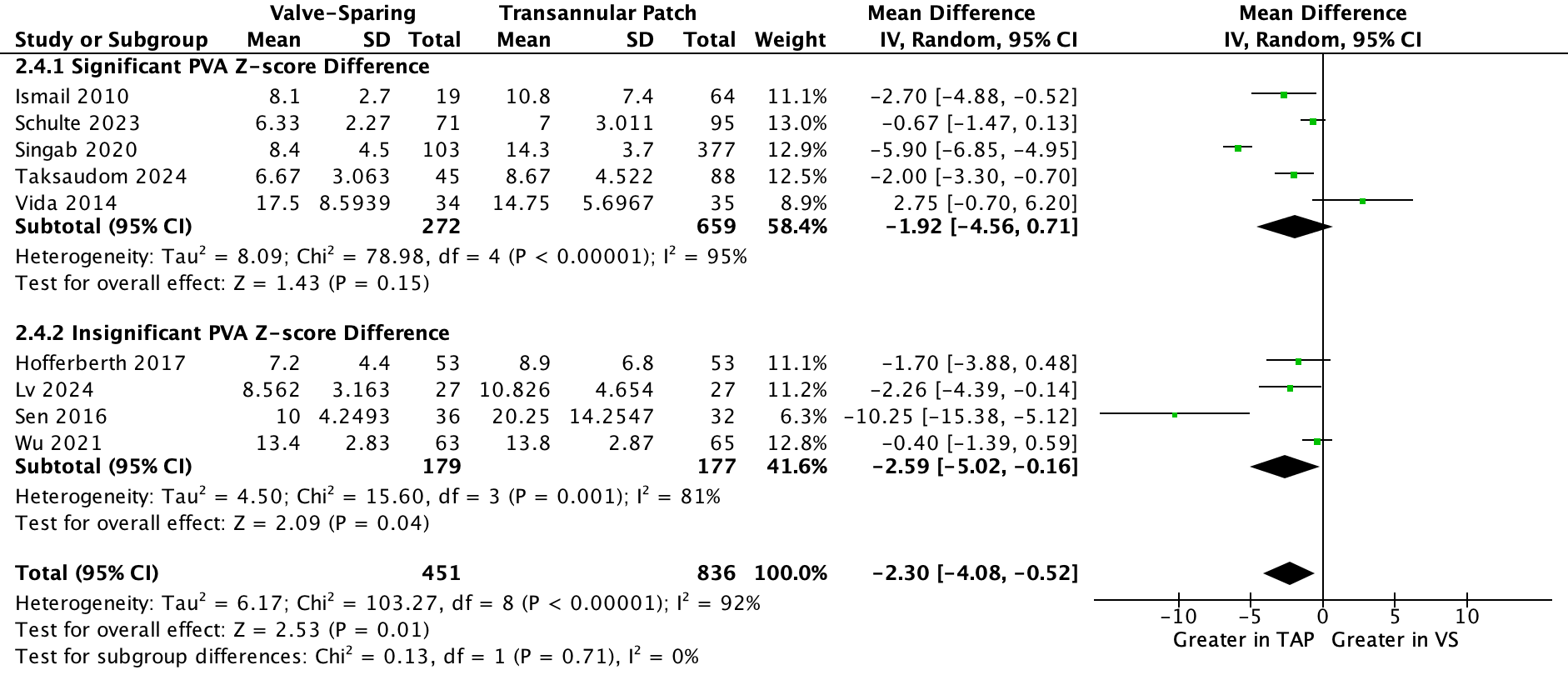
**

**Supplementary Figure 6.2.7: Extracorporeal Membrane Oxygenation (ECMO)**

**
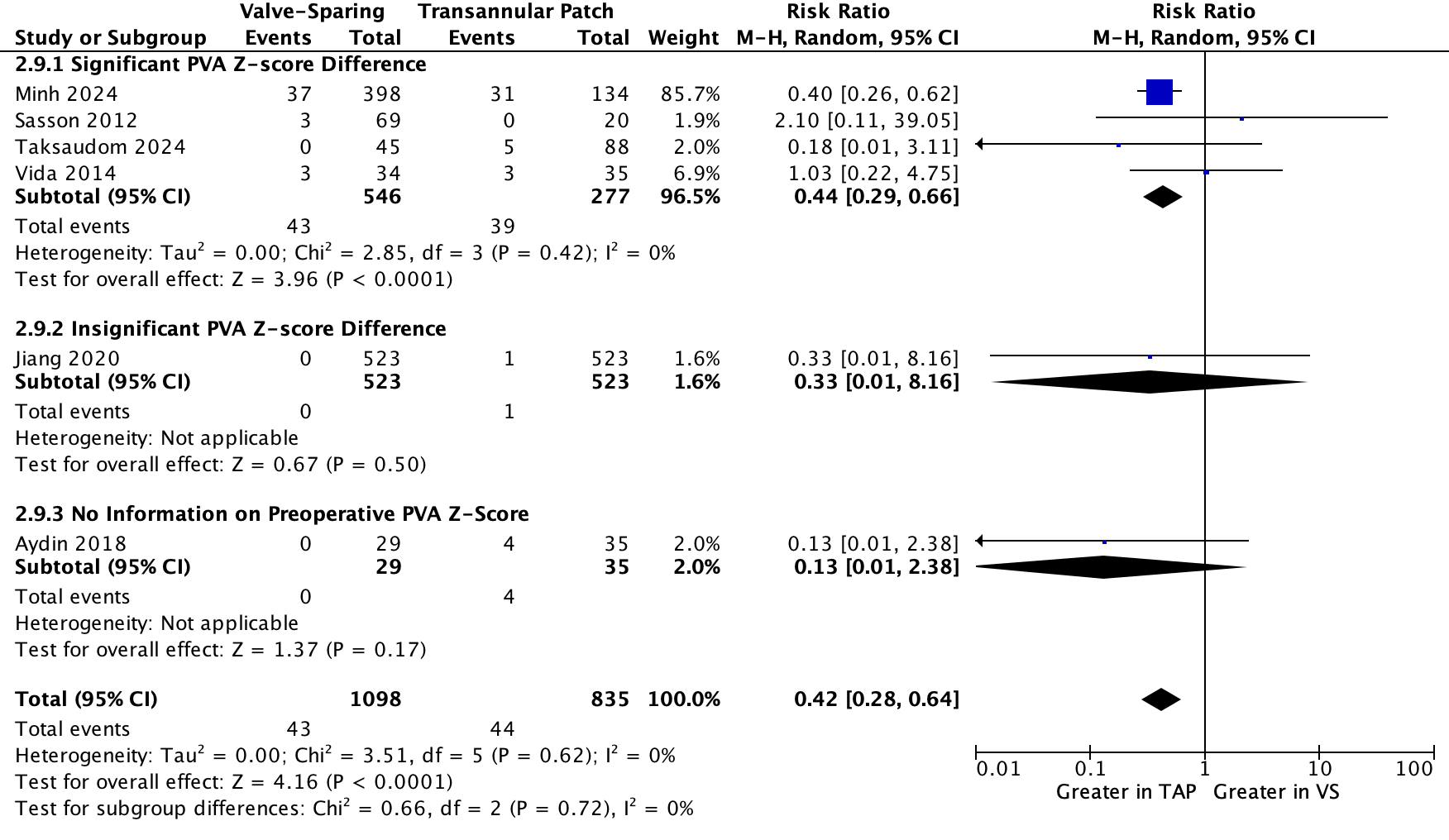
**

**Supplementary Figure 6.2.8: Inotropic Support Duration (Hours)**

**
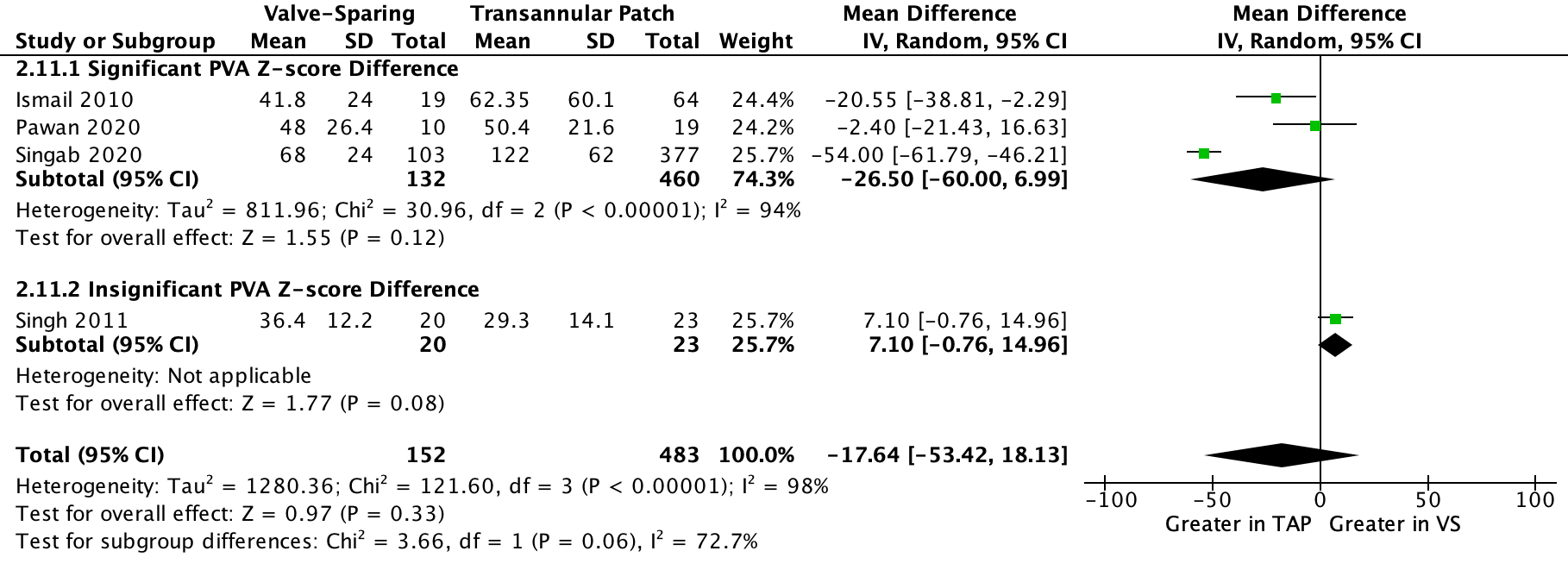
**

**Supplementary Figure 6.2.9: Pleural Effusion**

**
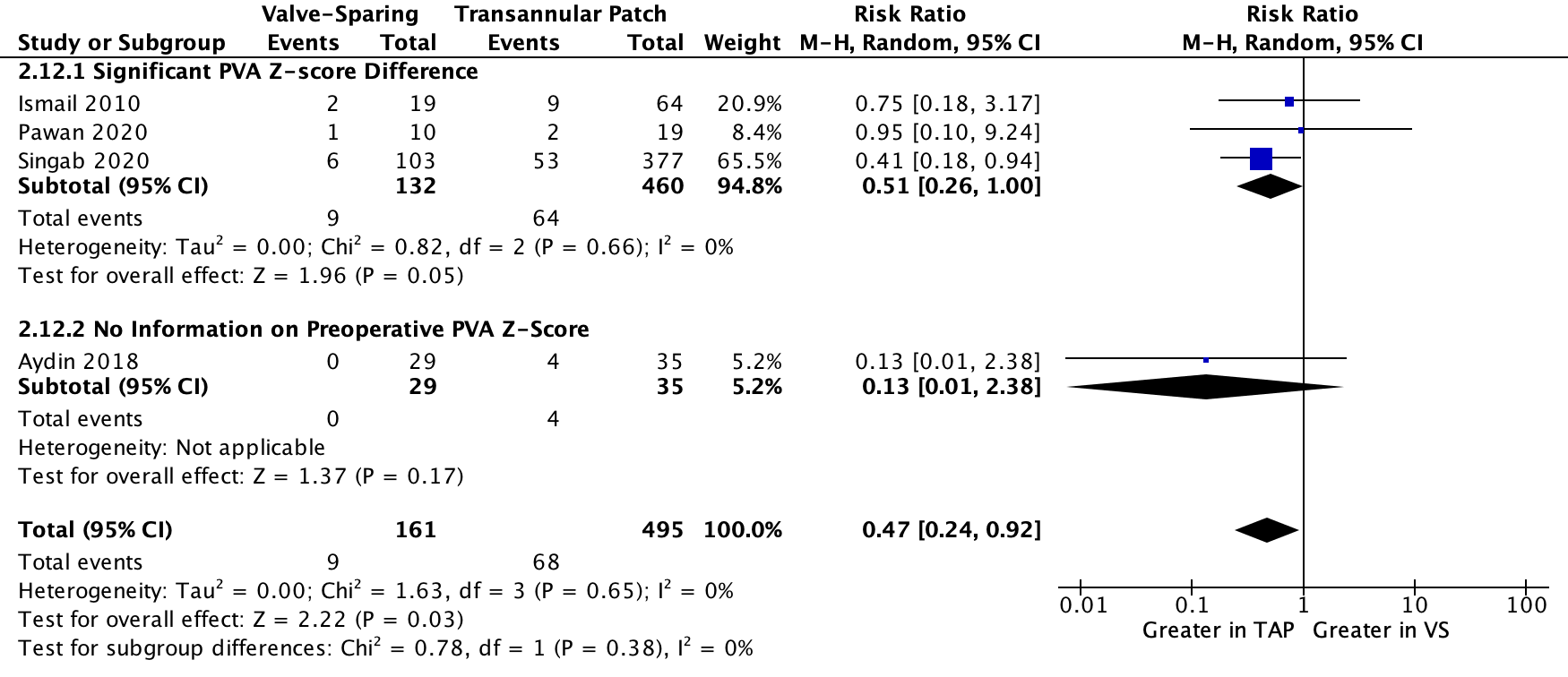
**

**Supplementary Figure 6.2.10: Acute Kidney Injury**

**
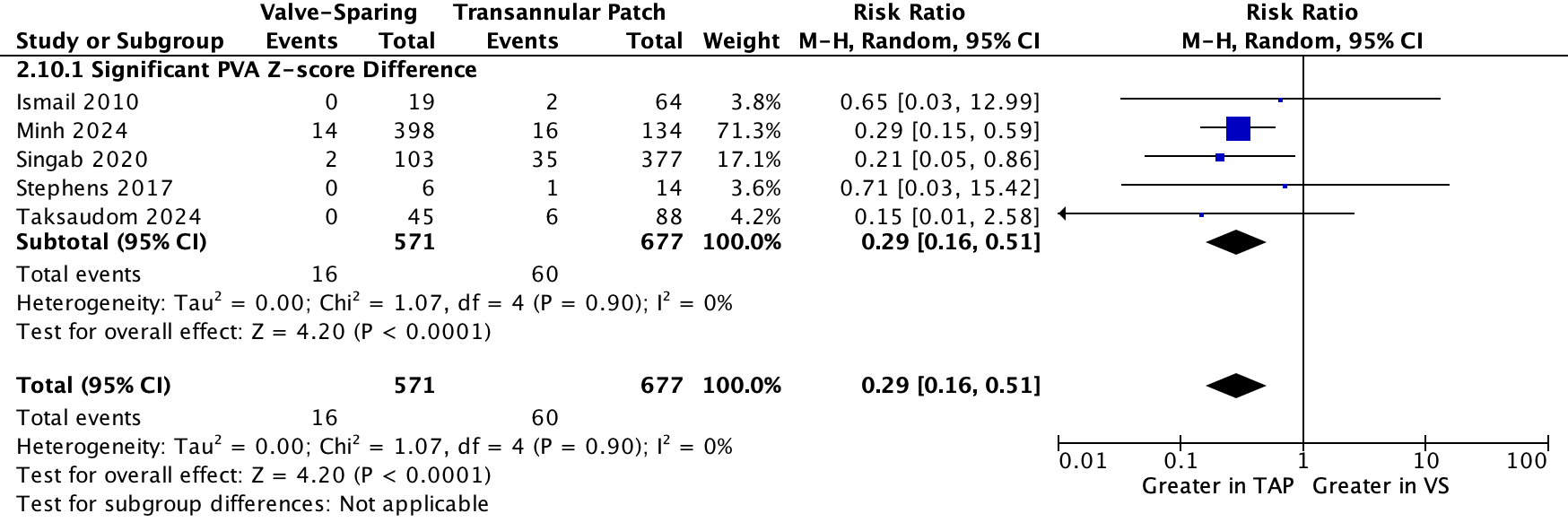
**

**Supplementary Figure 6.2.11: Neurological Complications**

**
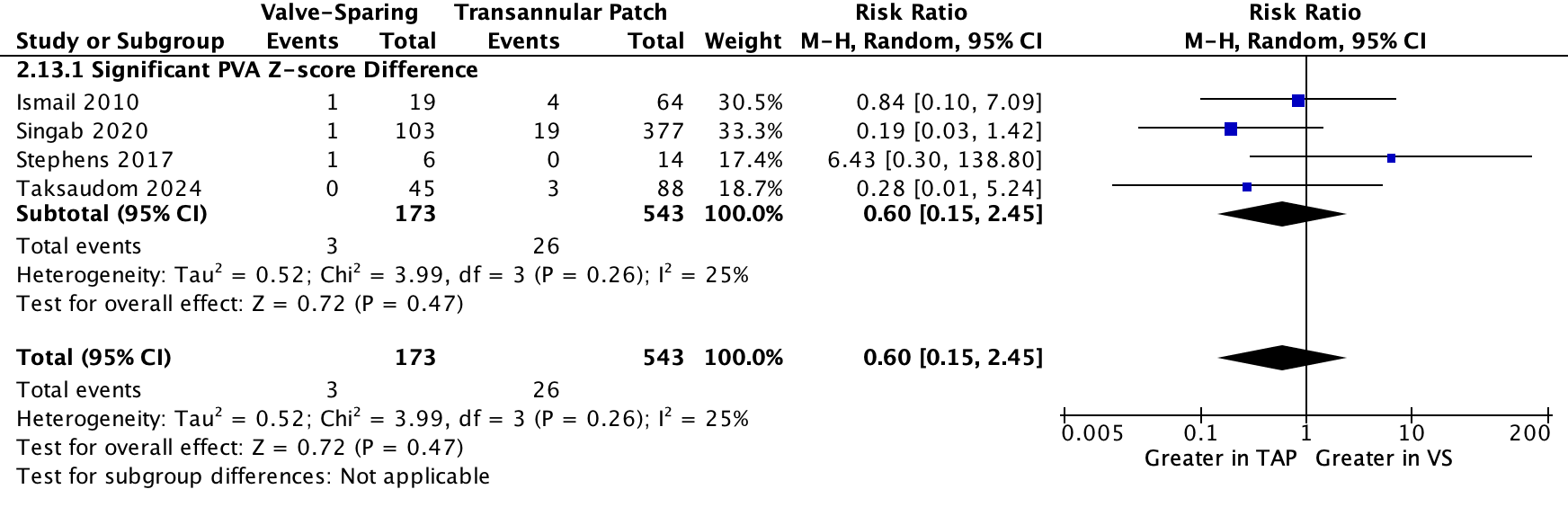
**

**Supplementary Figure 6.2.12: Chylothorax**

**
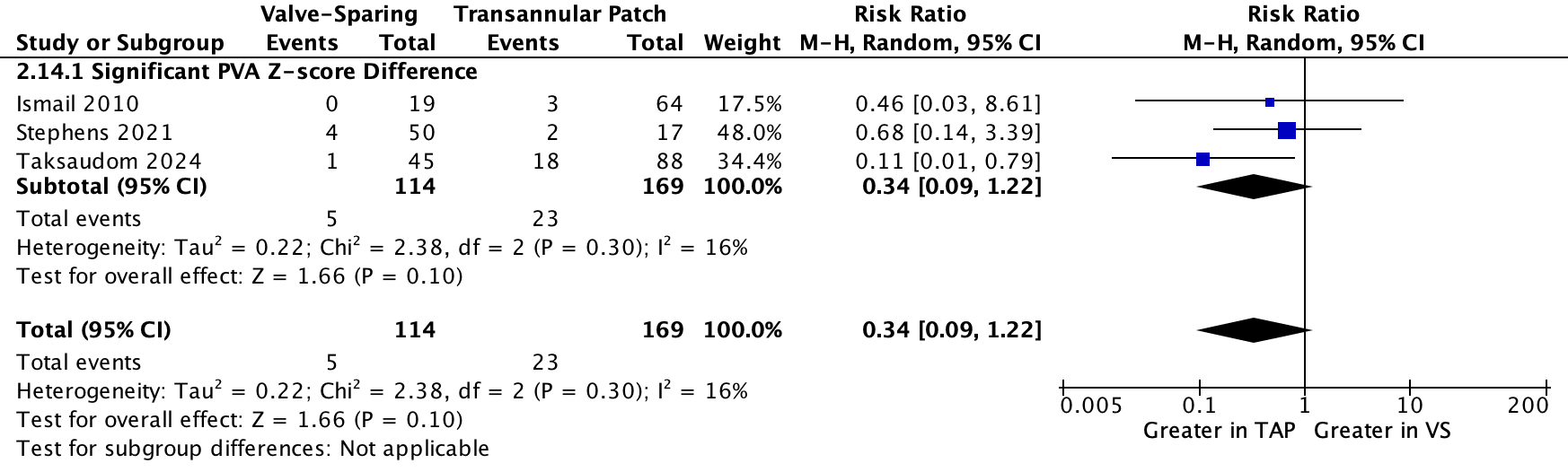
**

**Supplementary Figure 6.2.13: Pericardial Effusion**

**
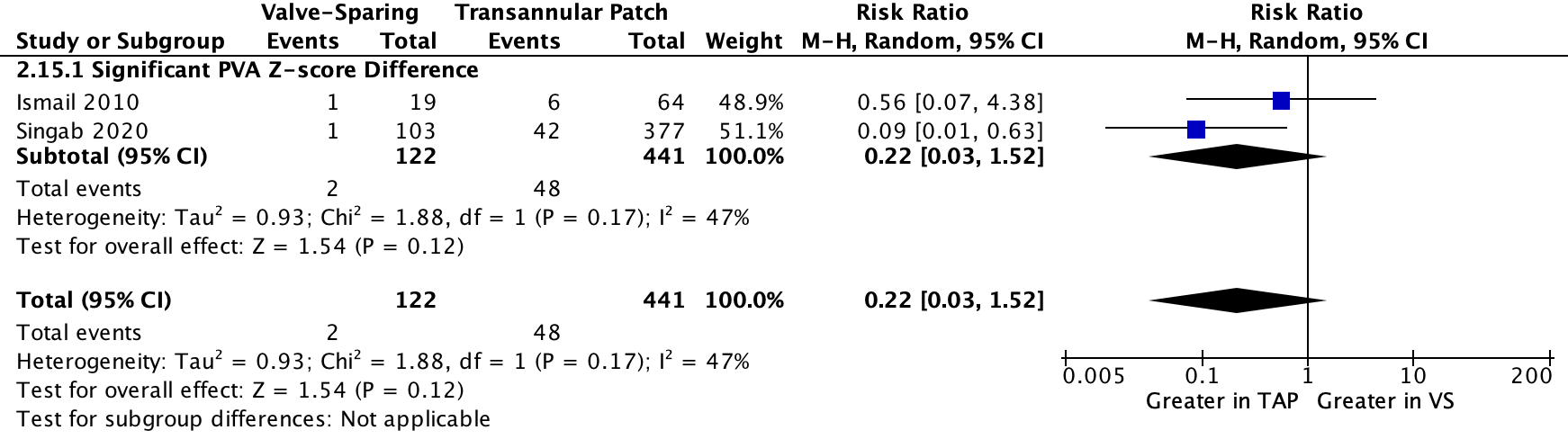
**

***Subsection 3: Postoperative Echocardiographic Outcomes***

**Supplementary Figure 6.3.1: Moderate/Severe Pulmonary Insufficiency**

**
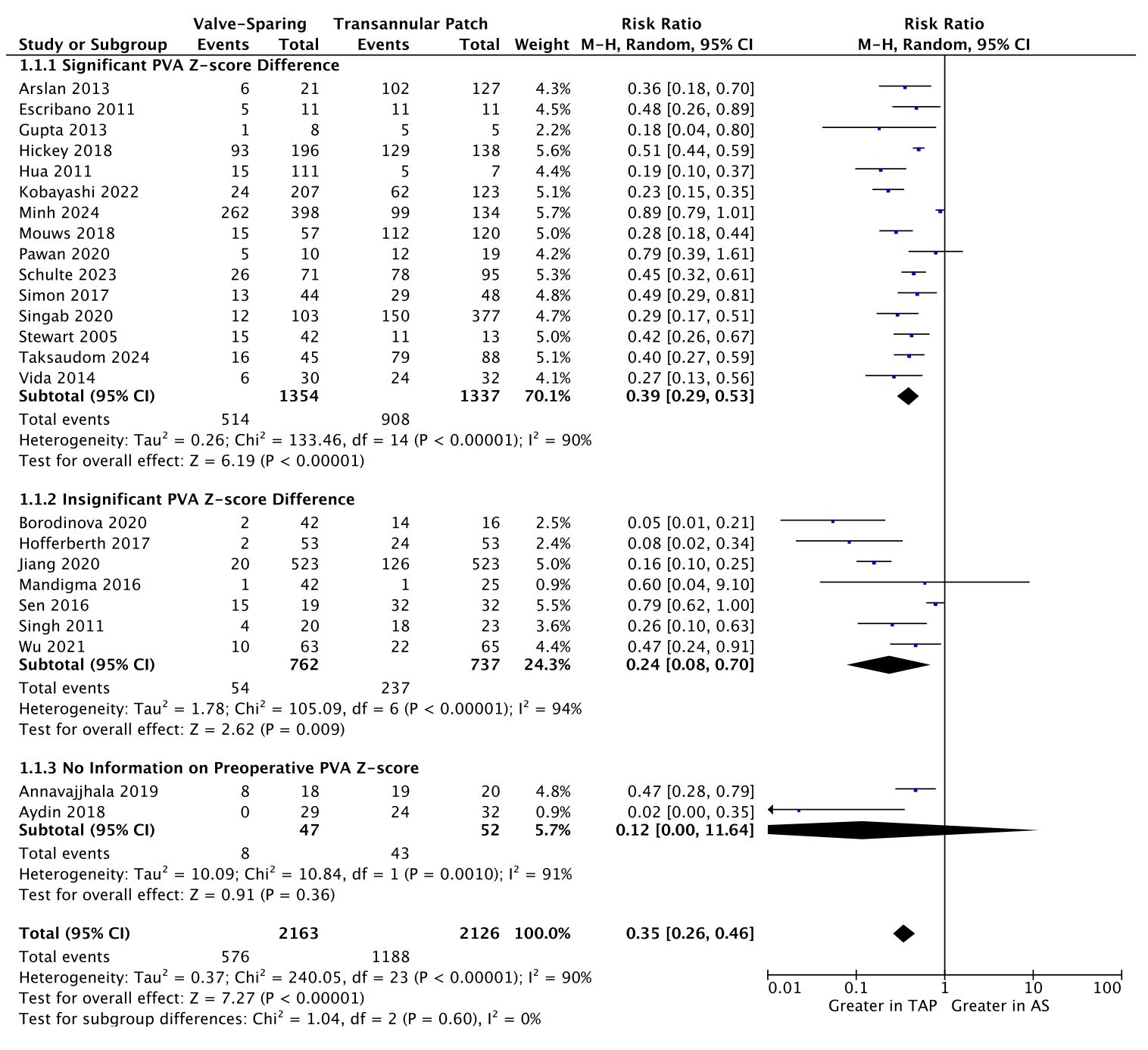
**

**Supplementary Figure 6.3.2: Right Ventricular (RV) Outflow Tract (RVOT) Pressure Gradient (mmHg)**

**
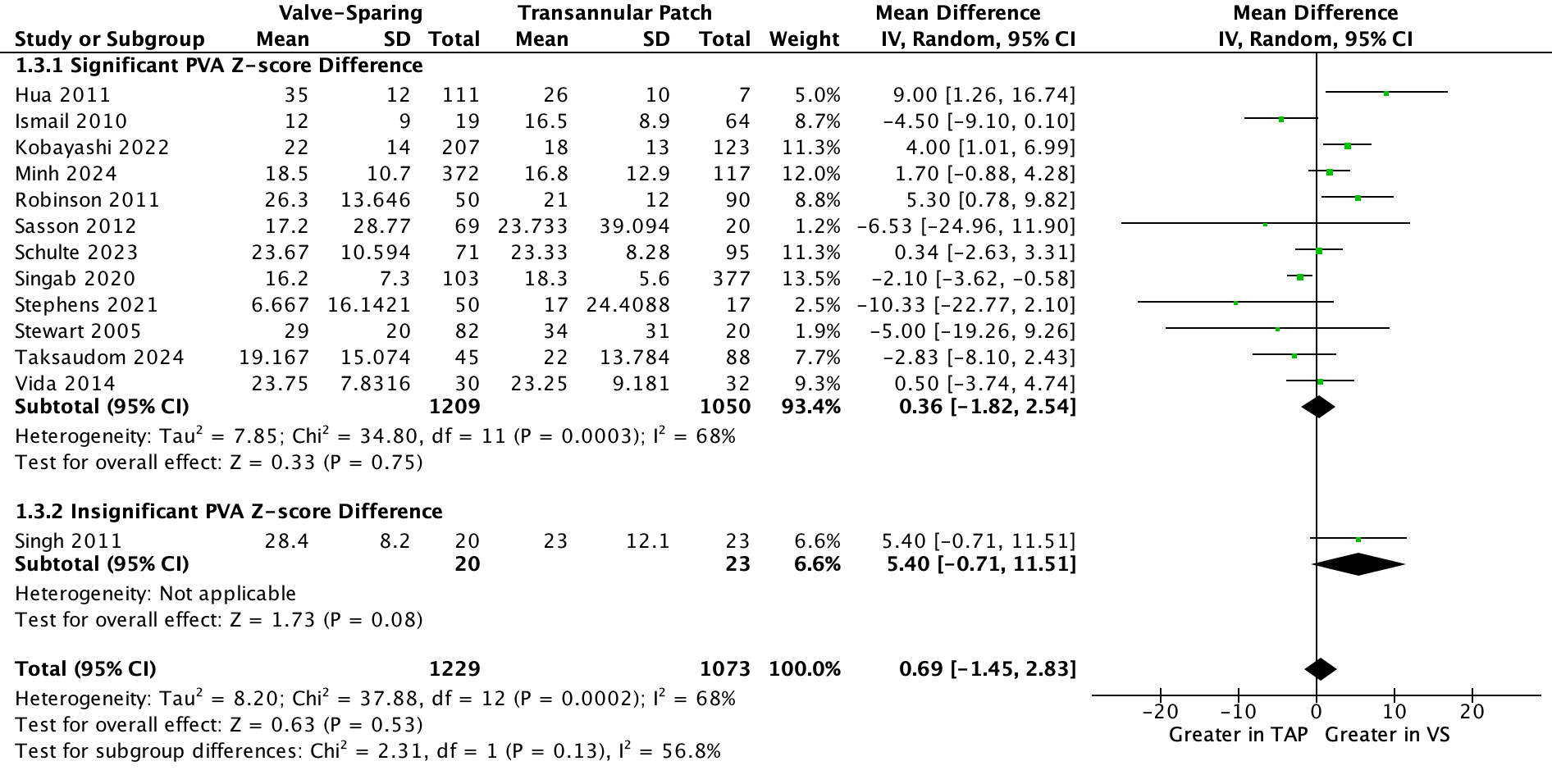
**

**Supplementary Figure 6.3.3: Pulmonary Valve Diameter Z-Score**

**
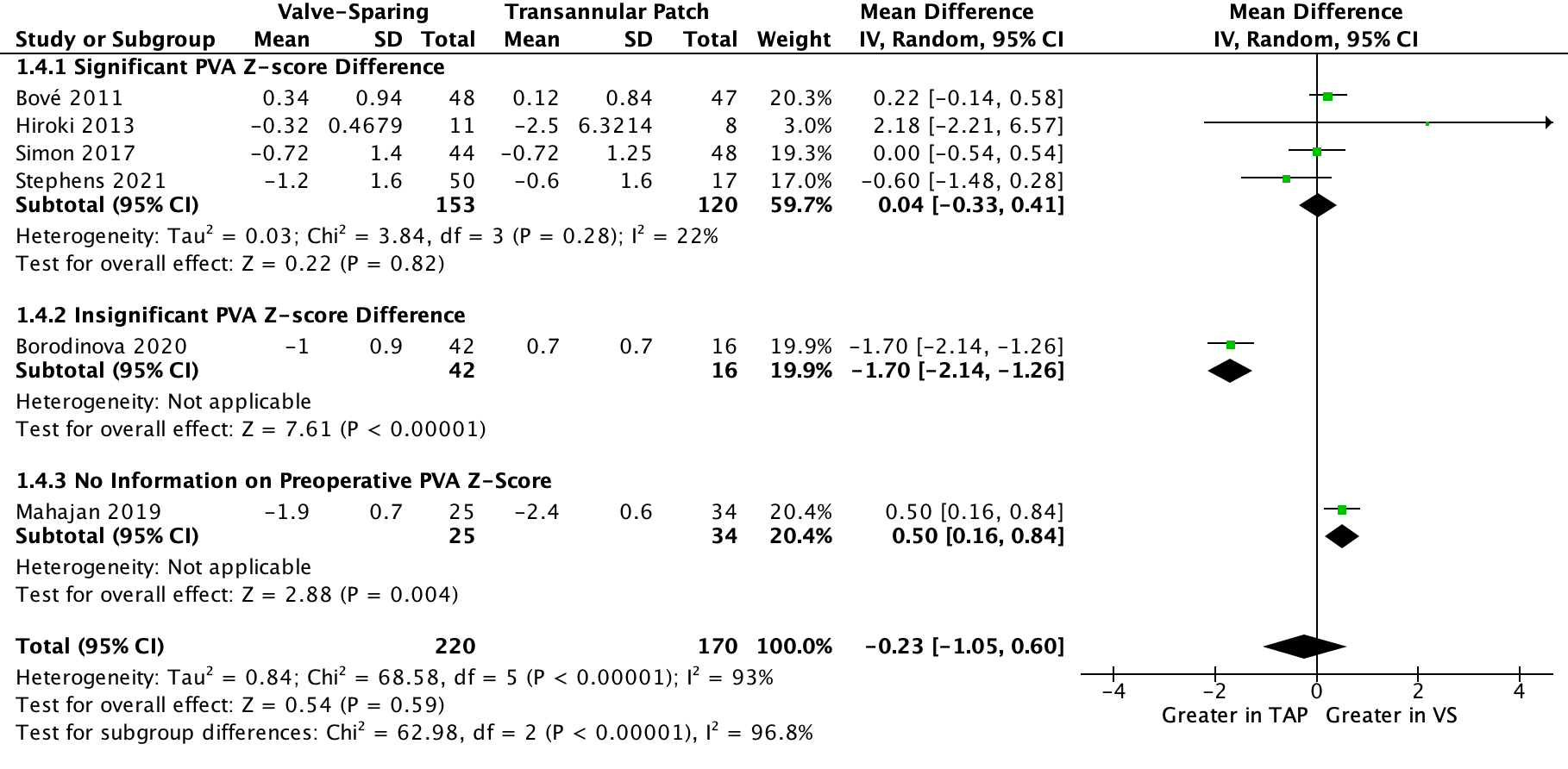
**

**Supplementary Figure 6.3.4: Residual RVOT Stenosis/Obstruction**

**
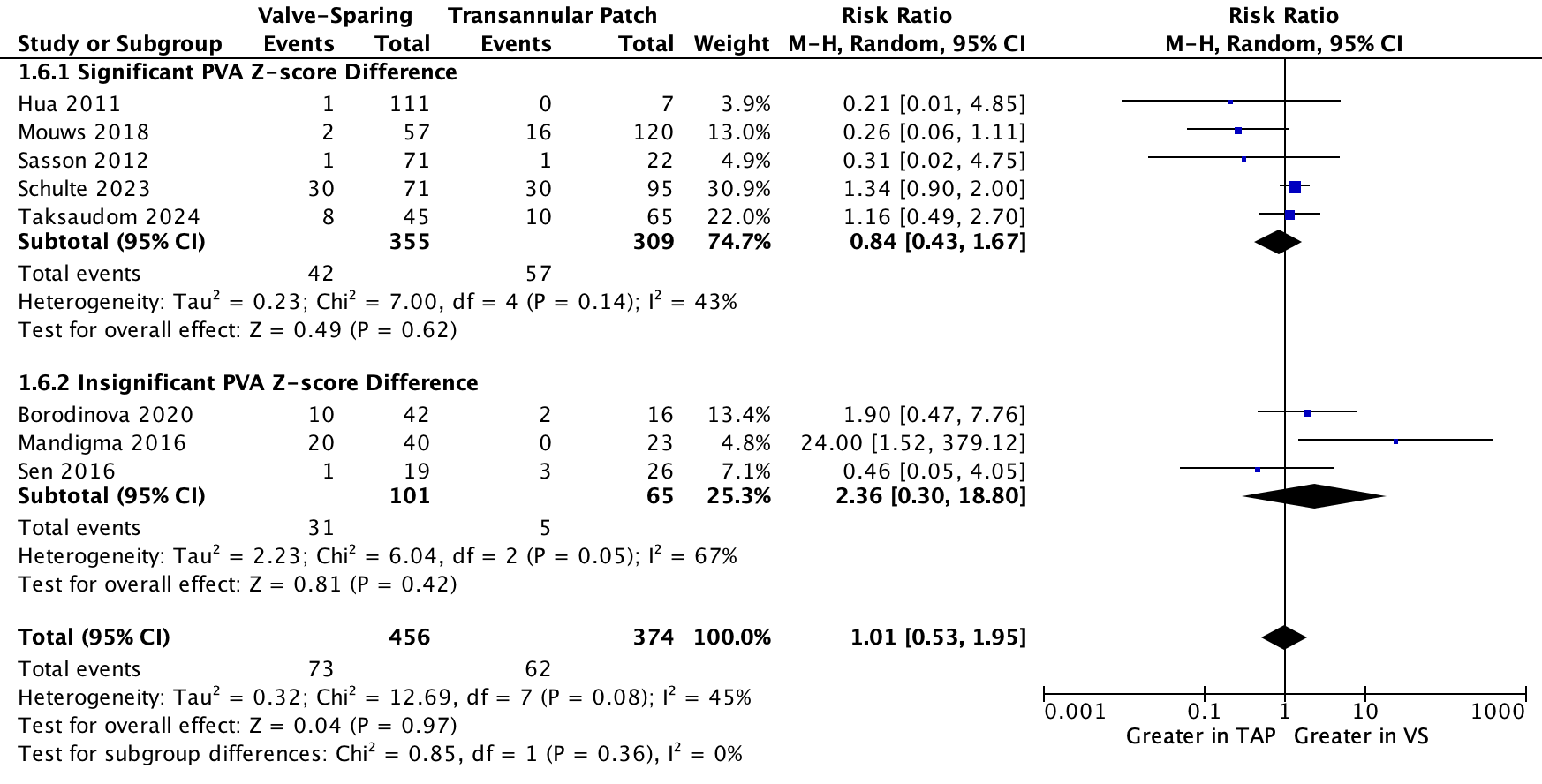
**

**Supplementary Figure 6.3.5: Tricuspid Valve Regurgitation**

**
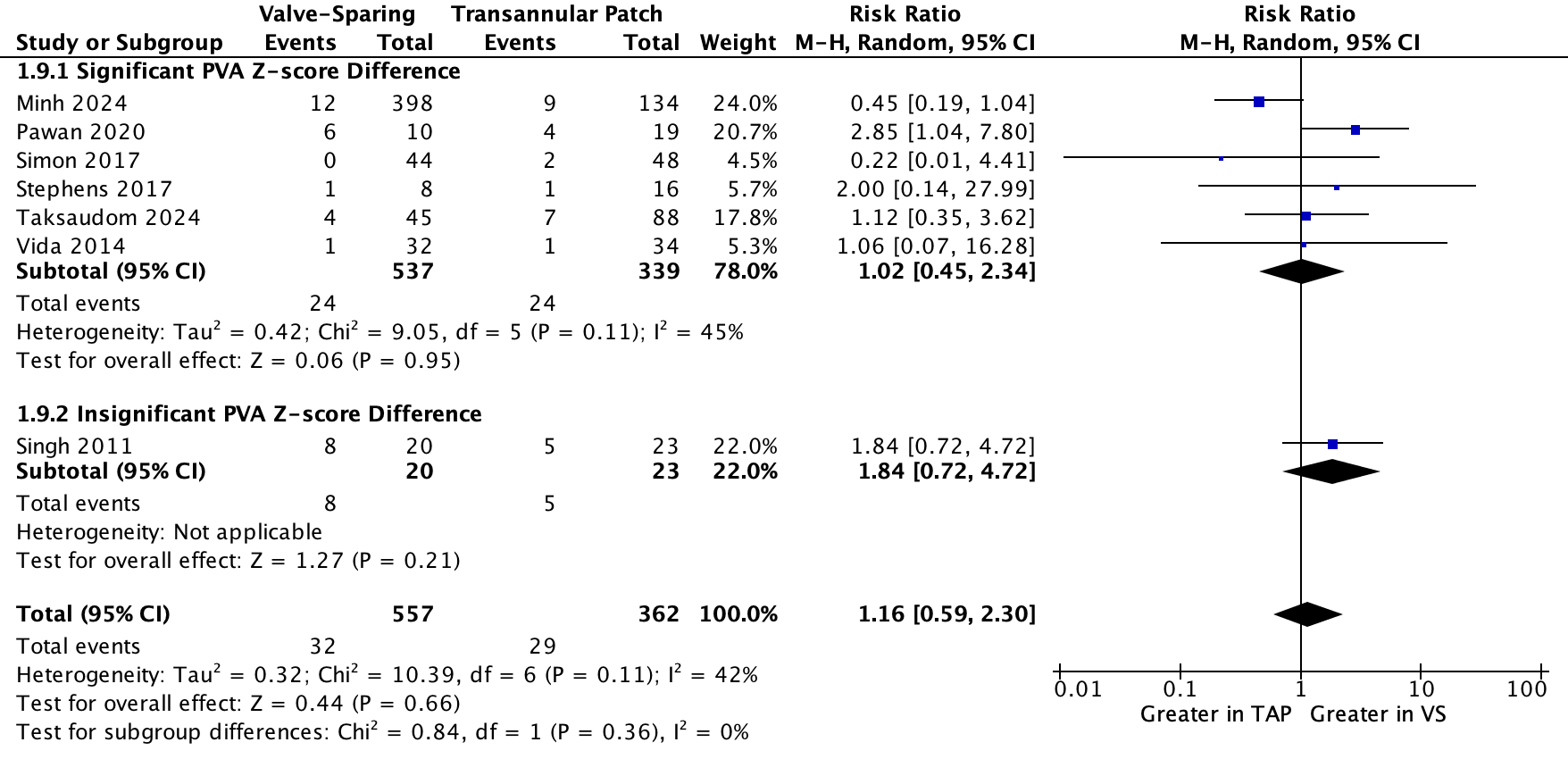
**

**Supplementary Figure 6.3.6: RV/LV Pressure Ratio**

**
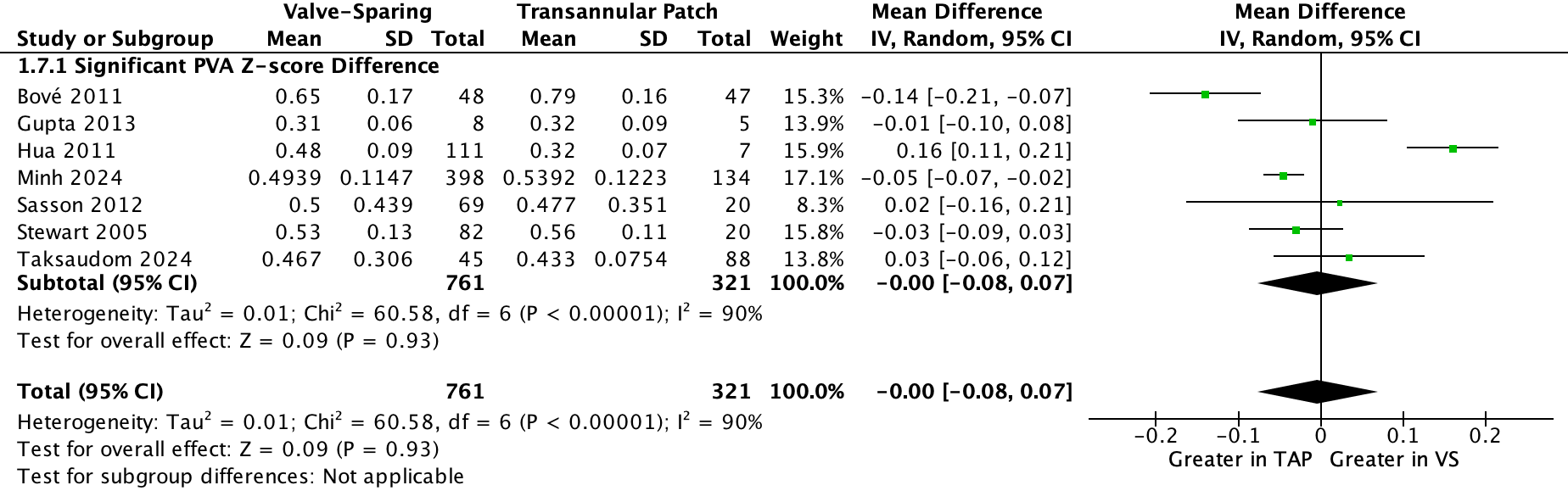
**

**Supplementary Figure 6.3.7: RV Systolic Dysfunction**

**
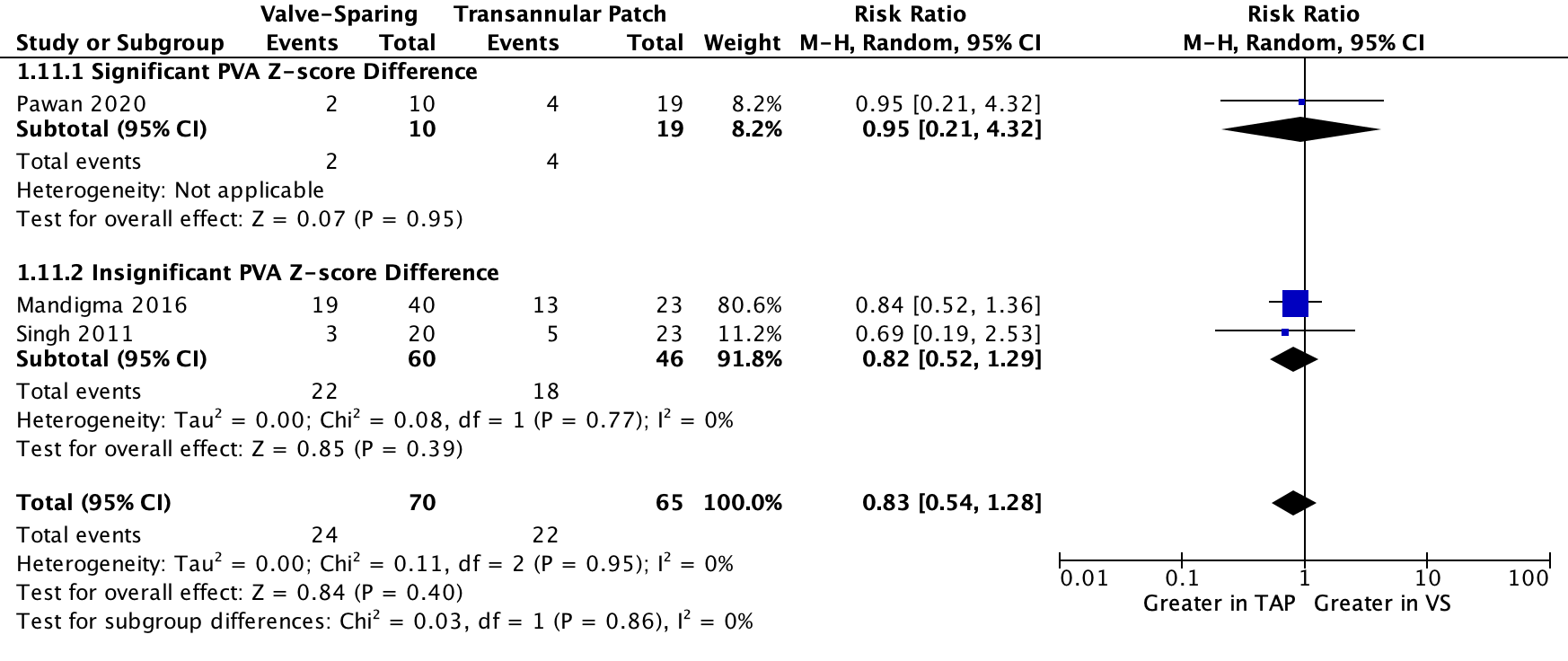
**

**Supplementary Figure 6.3.8: Maximum RVOT Velocity (m/s)**

**
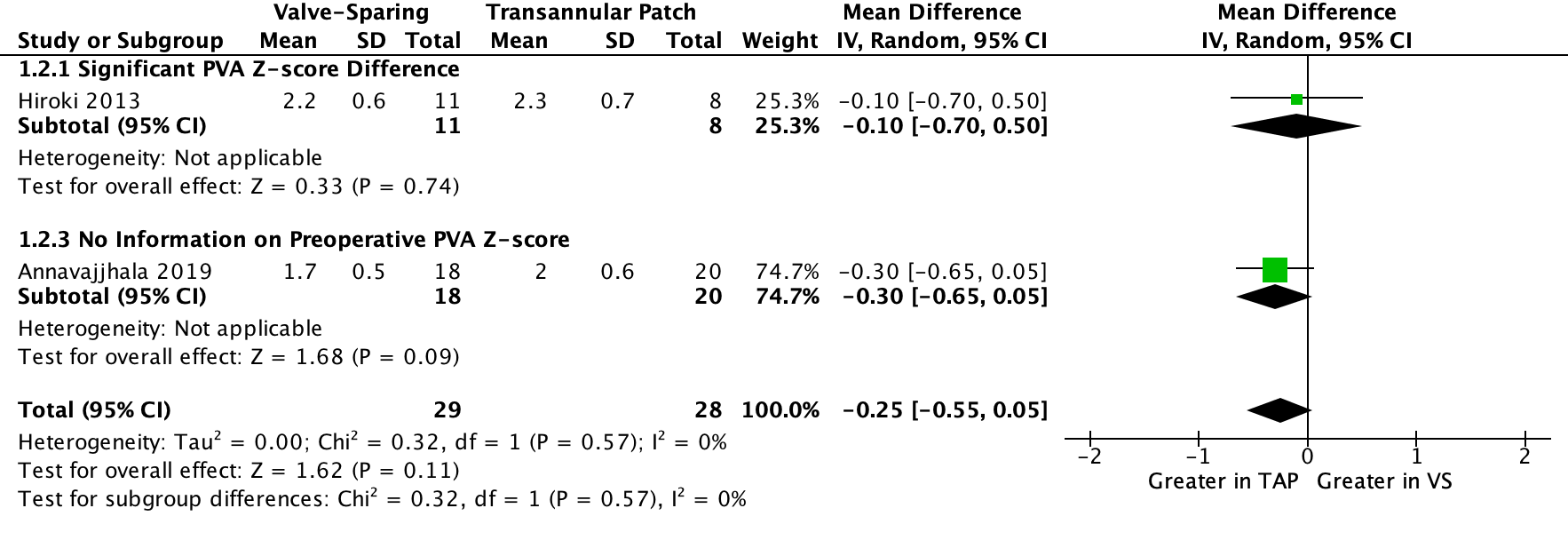
**

**Supplementary Figure 6.3.9: RVOT Dilation**

**
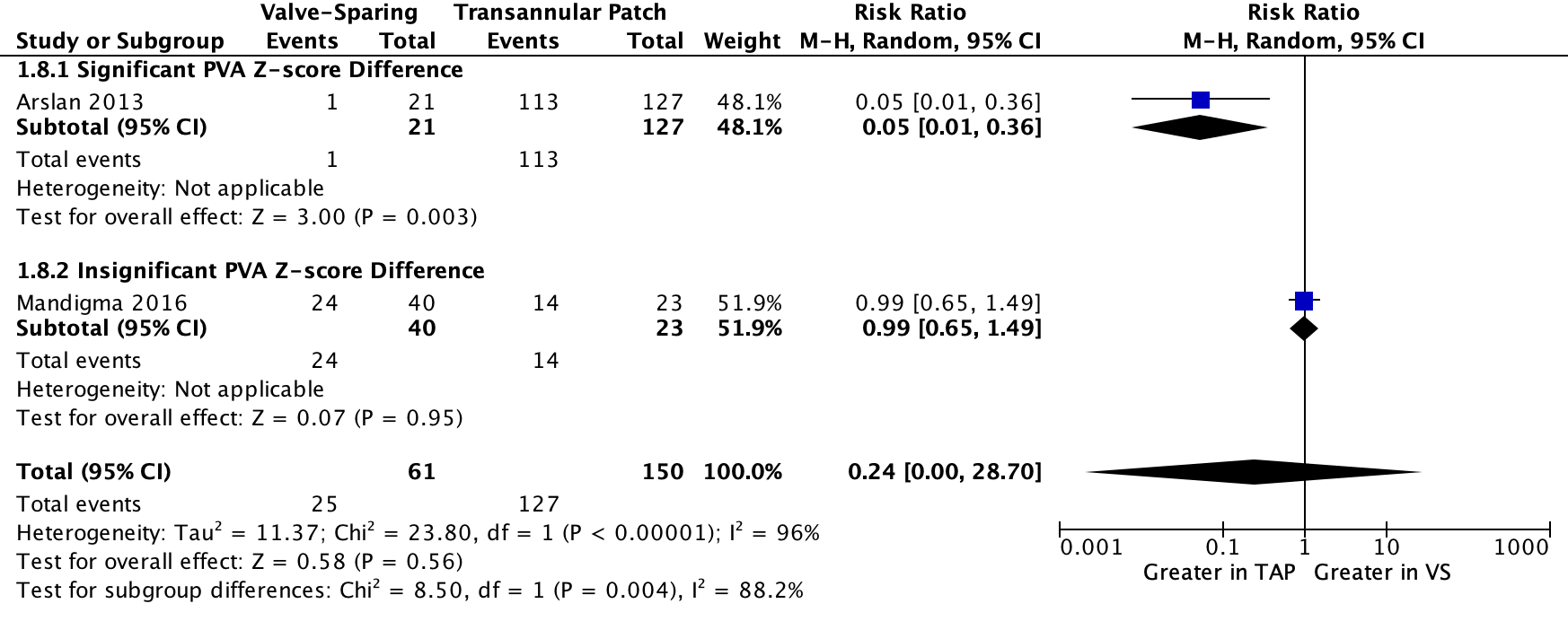
**

**Supplementary Figure 6.3.10: RV/LV Size Ratio**

**
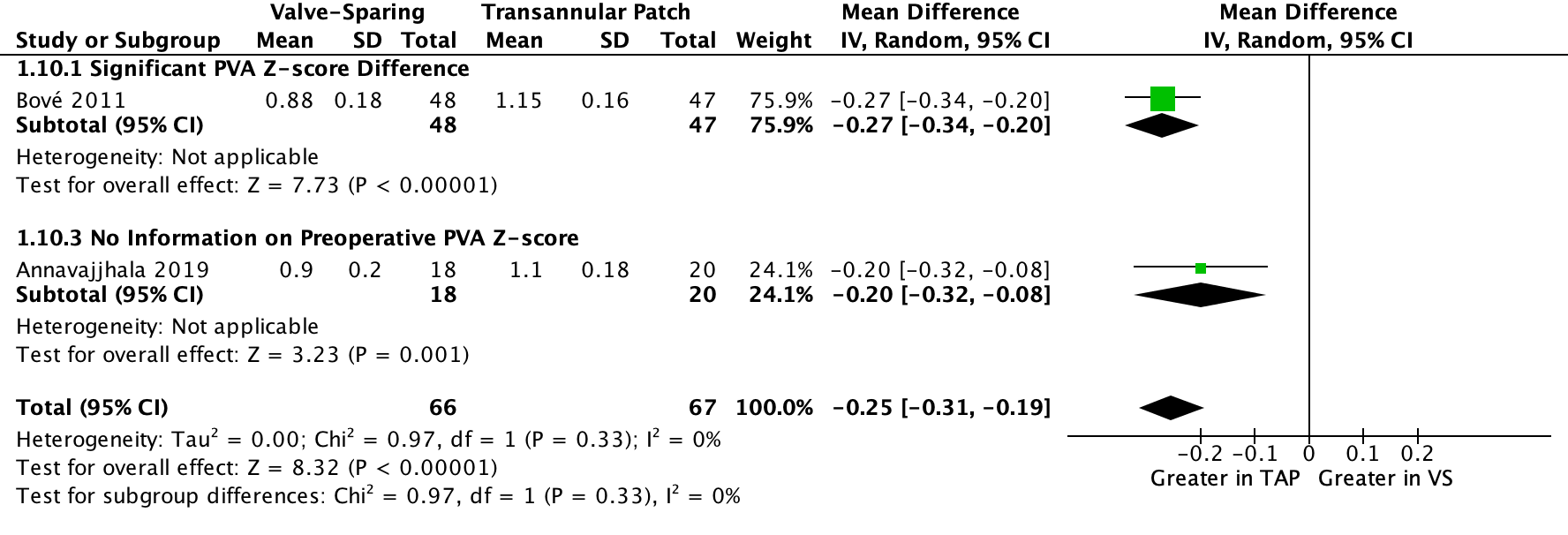
**

**Section 7: Bubble Plots for Meta-Regression**

For all the bubble plots below, the x-axis is the median follow-up for the included studies.

**Supplementary Figure 7.1: Pulmonary Insufficiency**

**
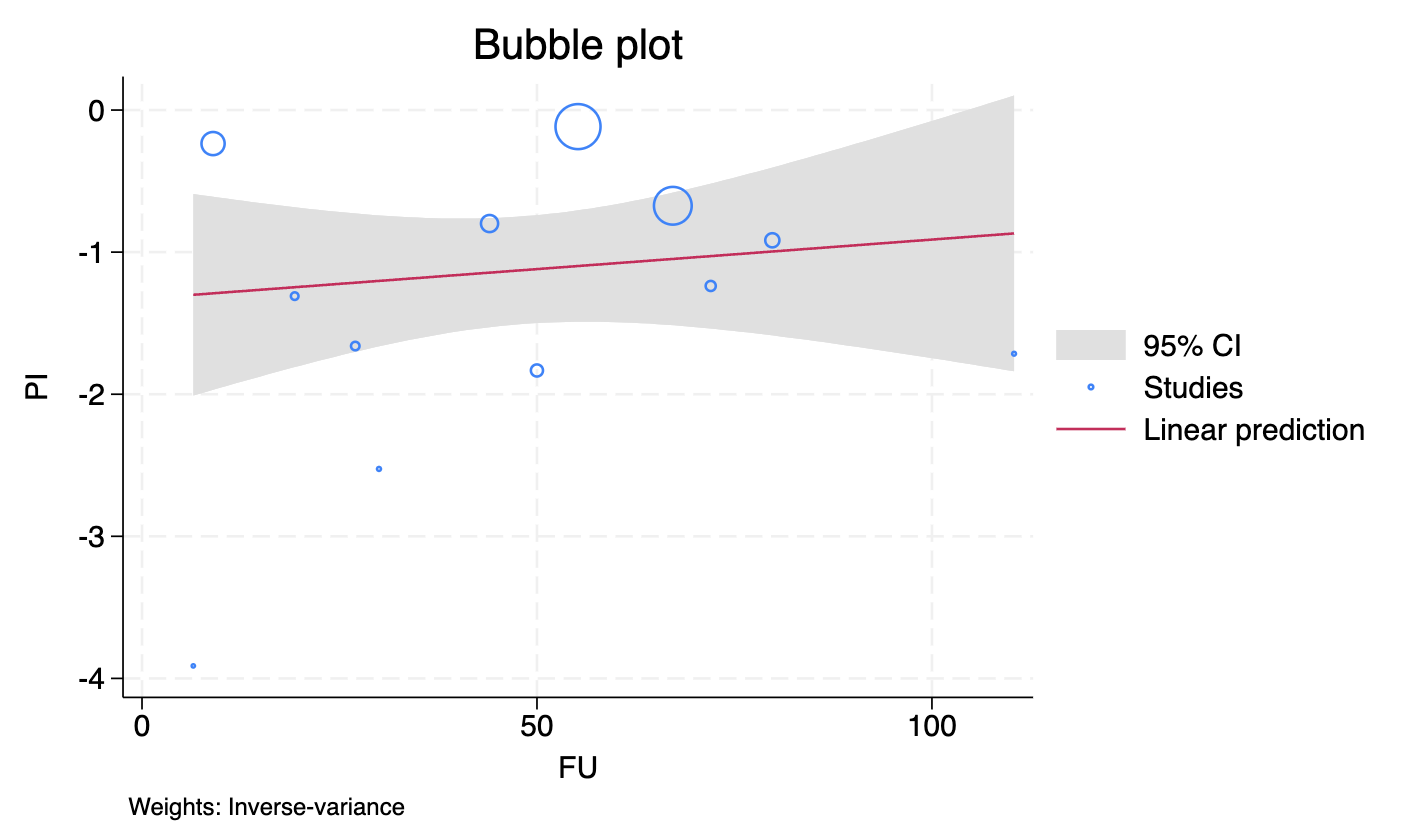
**

I^2^=92.53% and 𝜏^2^=0.3334 values describing residual heterogeneity after meta-regression were obtained. The regression coefficient for median follow-up was computed as –0.0102 (p = 0.571, R^2^=0).

**Supplementary Figure 7.2: Cardiovascular Reintervention Rate**

**
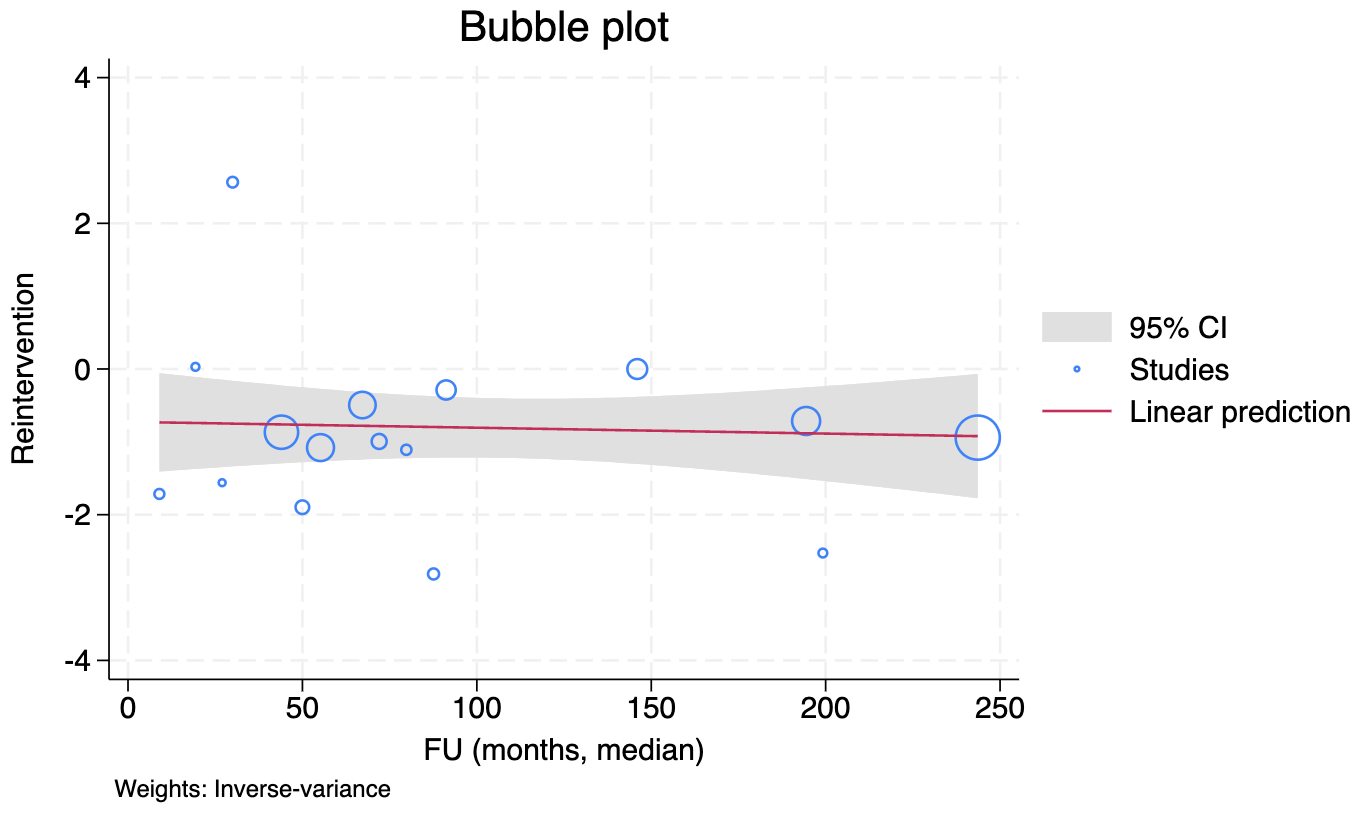
**

I^2^=43.0% and 𝜏^2^=0.2625 values describing residual heterogeneity after meta-regression were obtained. The regression coefficient for median follow-up was computed as –0.0008 (p=0.773, R^2^=0).

**Supplementary Figure 7.3: Overall Mortality**

**
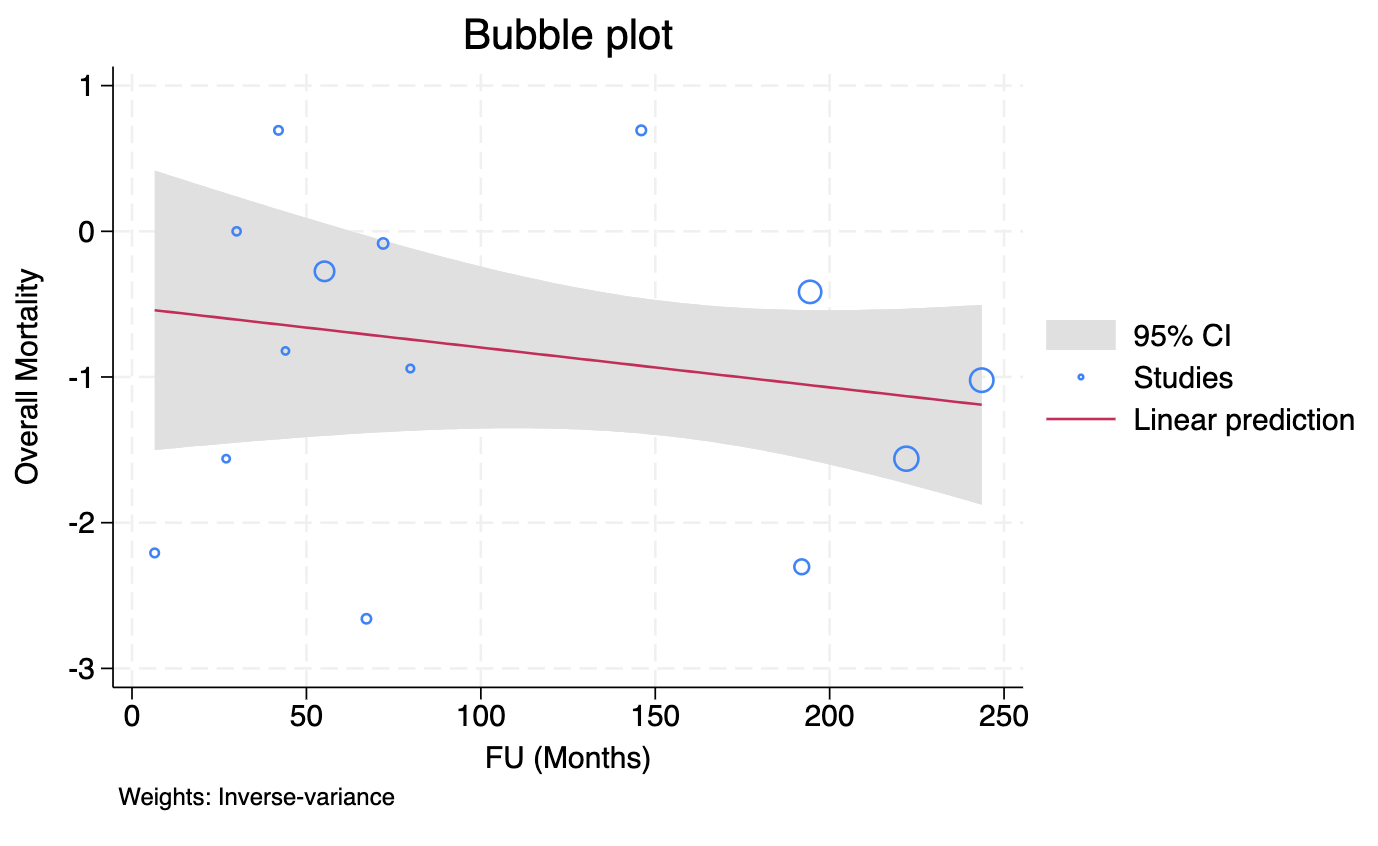
**

I^2^=9.40% and 𝜏^2^=0.0756 values describing residual heterogeneity after meta-regression were obtained. The regression coefficient for median follow-up was computed as –0.0027 (p=0.345, R^2^=0).

**Section 8: Risk of Bias Assessment**

**Supplementary Table:** Quality assessment of included observational studies using the ROBINS-I tool for non-randomized studies of intervention.

| **S. No** | **Study** | **ROBINS-I Domain** | | | | | | | |
| --- | --- | --- | --- | --- | --- | --- | --- | --- | --- |
|  |  | **Bias due to confounding** | **Bias in selection of participants into the study** | **Bias in classification of interventions** | **Bias due to deviations from intended interventions** | **Bias due to missing data** | **Bias in measurement of outcomes** | **Bias in selection of the reported result** | **Overall Bias** |
| 1 | Annavajjhala et al. (2019) | Moderate | Low | Low | Low | Moderate | Low | Low | Moderate |
| 2 | Arslan et al. (2013) | Moderate | Low | Low | Low | Low | Low | Low | Moderate |
| 3 | Aydin et al. (2018) | Moderate | Low | Low | Low | Moderate | Low | Low | Moderate |
| 4 | Blais et al. (2021) | Low | Low | Low | Low | Low | Low | Low | Low |
| 5 | Bonura et al. (2023) | Serious | Moderate | Low | Moderate | Low | Low | Moderate | Serious |
| 6 | Borodinova et al. (2019) | Low | Low | Low | Low | Low | Low | Low | Low |
| 7 | Bové et al. (2011) | Moderate | Low | Low | Low | Low | Low | Low | Moderate |
| 8 | Escribano et al. (2011) | Serious | Low | Low | Low | Low | Low | Low | Serious |
| 9 | Gupta et al. (2012) | Moderate | Low | Low | Low | Low | Low | Low | Moderate |
| 10 | Hickey et al. (2018) | Moderate | Low | Low | Low | Low | Low | Low | Moderate |
| 11 | Hiroki Ito et al. (2013) | Serious | Low | Low | Low | Low | Low | Low | Serious |
| 12 | Hofferberth et al. (2017) | Low | Low | Low | Low | Low | Low | Low | Low |
| 13 | Hua et al. (2011) | Serious | Low | Low | Low | Low | Low | Low | Serious |
| 14 | Ismail et al. (2010) | Moderate | Low | Low | Low | Low | Low | Low | Moderate |
| 15 | Jiang et al. (2020) | Low | Low | Low | Low | Low | Low | Low | Low |
| 16 | Kim et al. (2014) | Low | Low | Low | Low | Low | Low | Low | Low |
| 17 | Kobayashi et al. (2022) | Moderate | Low | Low | Low | Low | Low | Low | Moderate |
| 18 | Lv et al. (2022) | Low | Low | Low | Low | Low | Low | Low | Low |
| 19 | Mahajan et al. (2019) | Serious | Low | Low | Low | Low | Low | Low | Serious |
| 20 | Mandigma et al. (2016) | Low | Low | Low | Low | Low | Low | Low | Low |
| 21 | Minh et al. (2024) | Moderate | Low | Low | Low | Low | Low | Low | Moderate |
| 22 | Mouws et al. (2018) | Moderate | Low | Low | Low | Low | Low | Low | Moderate |
| 23 | Ono et al. (2022) | Low | Low | Low | Low | Low | Low | Low | Low |
| 24 | Padalino et al. (2017) | Moderate | Low | Low | Low | Low | Low | Low | Moderate |
| 25 | Pawan et al. (2020) | Serious | Low | Low | Low | Moderate | Low | Low | Serious |
| 26 | Robinson et al. (2011) | Serious | Low | Low | Low | Low | Low | Low | Serious |
| 27 | Sasson et al. (2012) | Serious | Low | Low | Low | Moderate | Low | Low | Serious |
| 28 | Schulte et al. (2023) | Serious | Moderate | Low | Low | Low | Low | Moderate | Serious |
| 29 | Sen et al. (2016) | Low | Low | Low | Low | Low | Low | Low | Low |
| 30 | Simon et al. (2017) | Serious | Low | Low | Low | Low | Low | Low | Serious |
| 31 | Singab et al. (2020) | Serious | Low | Low | Low | Low | Low | Low | Serious |
| 32 | Singh et al. (2011) | Low | Low | Low | Low | Low | Low | Low | Low |
| 33 | Smith et al. (2018) | Moderate | Low | Low | Low | Low | Low | Low | Moderate |
| 34 | Stephens et al. (2017) | Serious | Low | Low | Low | Low | Low | Low | Serious |
| 35 | Stephens et al. (2021) | Serious | Low | Low | Low | Low | Low | Low | Serious |
| 36 | Stewart et al. (2005) | Serious | Low | Low | Low | Low | Low | Low | Serious |
| 37 | Taksaudom et al. (2024) | Low | Low | Low | Low | Low | Low | Low | Low |
| 38 | Van den Bosch et al. (2019) | Moderate | Low | Low | Low | Low | Low | Low | Moderate |
| 39 | Vida et al. (2014) | Serious | Low | Low | Low | Low | Low | Low | Serious |
| 40 | Wu et al. (2021) | Low | Low | Low | Low | Low | Low | Low | Low |

**Section 9: GRADE Certainty of Evidence Assessment**

**Author(s):**

**Question:** Valve-Sparing Repair compared to Transannular Patch Repair for Tetralogy of Fallot

**Setting:** Echocardiographic Outcomes

| **Certainty assessment** | | | | | | | **№ of patients** | | **Effect** | | **Certainty** | **Importance** |
| --- | --- | --- | --- | --- | --- | --- | --- | --- | --- | --- | --- | --- |
| **№ of studies** | **Study design** | **Risk of bias** | **Inconsistency** | **Indirectness** | **Imprecision** | **Other considerations** | **Valve-Sparing Repair** | **Transannular Patch Repair** | **Relative (95% CI)** | **Absolute (95% CI)** |  |  |
| **Post-Operative PV Regurgitation** | | | | | | | | | | | | |
| 24 | non-randomised studies | serious^a^ | not serious | not serious | not serious | strong association | 576/2163 (26.6%) | 1188/2126 (55.9%) | **RR 0.35** (0.26 to 0.46) | **363 fewer per 1,000** (from 414 fewer to 302 fewer) | ⨁⨁⨁⨁ High |  |
| **Post-Operative Maximum RVOT Velocity** | | | | | | | | | | | | |
| 2 | non-randomised studies | very serious^b^ | not serious | not serious | not serious | none | 29 | 28 | - | MD **0.25 lower** (0.55 lower to 0.05 higher) | ⨁◯◯◯ Very low |  |
| **Post-Operative RVOT Pressure Gradient** | | | | | | | | | | | | |
| 13 | non-randomised studies | serious^a^ | not serious | not serious | not serious | none | 1229 | 1073 | - | MD **0.69 higher** (1.45 lower to 2.83 higher) | ⨁◯◯◯ Very low |  |
| **Post-Operative Pulmonary Valve Diameter Z-Score** | | | | | | | | | | | | |
| 6 | non-randomised studies | serious^a^ | very serious^c^ | not serious | not serious | none | 220 | 170 | - | MD **0.23 lower** (1.05 lower to 0.6 higher) | ⨁◯◯◯ Very low |  |
| **Post-Operative Residual RVOT Stenosis** | | | | | | | | | | | | |
| 8 | non-randomised studies | serious^a^ | not serious | not serious | not serious | none | 73/456 (16.0%) | 62/374 (16.6%) | **RR 1.01** (0.53 to 1.95) | **2 more per 1,000** (from 78 fewer to 157 more) | ⨁◯◯◯ Very low |  |
| **Post-Operative RV/LV Pressure Ratio** | | | | | | | | | | | | |
| 7 | non-randomised studies | serious^a^ | not serious | not serious | not serious | none | 761 | 321 | - | MD **0**  (0.08 lower to 0.07 higher) | ⨁◯◯◯ Very low |  |
| **Post-Operative RVOT Dilation** | | | | | | | | | | | | |
| 2 | non-randomised studies | not serious | not serious | not serious | very serious^d^ | strong association | 25/61 (41.0%) | 127/150 (84.7%) | **RR 0.24** (0.00 to 28.70) | **643 fewer per 1,000** (from -- to 1,000 more) | ⨁◯◯◯ Very low |  |
| **Post-Operative TV Regurgitation** | | | | | | | | | | | | |
| 7 | non-randomised studies | serious^b^ | not serious | not serious | not serious | none | 32/557 (5.7%) | 29/362 (8.0%) | **RR 1.16** (0.59 to 2.30) | **13 more per 1,000** (from 33 fewer to 104 more) | ⨁◯◯◯ Very low |  |
| **Post-Operative RV/LV Size Ratio** | | | | | | | | | | | | |
| 2 | non-randomised studies | serious^e^ | not serious | not serious | not serious | none | 66 | 67 | - | MD **0.25 lower** (0.31 lower to 0.19 lower) | ⨁⨁⨁◯ Moderate |  |
| **Post-Operative RV Systolic Dysfunction** | | | | | | | | | | | | |
| 3 | non-randomised studies | not serious | not serious | not serious | not serious | none | 24/70 (34.3%) | 22/65 (33.8%) | **RR 0.83** (0.54 to 1.28) | **58 fewer per 1,000** (from 156 fewer to 95 more) | ⨁⨁◯◯ Low |  |

**CI:** confidence interval; **MD:** mean difference; **RR:** risk ratio

#### Explanations

a. A majority of studies had a moderate/serious risk of bias due to lack of control for confounding. This was, however, offset by the subgroup analysis that was performed for subgroup B.

b. Only studies rated as moderate/serious risk of bias were included in this outcome.

c. I^2 > 75%

d. Very wide confidence interval.

e. Only studies rated as moderate risk of bias were included in this outcome.

**Author(s):**

**Question:** Valve-Sparing Repair compared to Transannular Patch Repair for Tetralogy of Fallot

**Setting:** Clinical Outcomes

| **Certainty assessment** | | | | | | | **№ of patients** | | **Effect** | | **Certainty** | **Importance** |
| --- | --- | --- | --- | --- | --- | --- | --- | --- | --- | --- | --- | --- |
| **№ of studies** | **Study design** | **Risk of bias** | **Inconsistency** | **Indirectness** | **Imprecision** | **Other considerations** | **Valve-Sparing Repair** | **Transannular Patch Repair** | **Relative (95% CI)** | **Absolute (95% CI)** |  |  |
| **Ventilation Duration (Hours)** | | | | | | | | | | | | |
| 9 | non-randomised studies | serious^a^ | very serious^b^ | not serious | serious^c^ | none | 721 | 829 | - | MD **15.33 lower** (30.2 lower to 0.46 lower) | ⨁◯◯◯ Very low |  |
| **Post-Operative Arrythmia** | | | | | | | | | | | | |
| 10 | non-randomised studies | serious^a^ | not serious | not serious | not serious | none | 106/1313 (8.1%) | 185/1761 (10.5%) | **RR 0.73** (0.58 to 0.92) | **28 fewer per 1,000** (from 44 fewer to 8 fewer) | ⨁◯◯◯ Very low |  |
| **Length of ICU Stay (Days)** | | | | | | | | | | | | |
| 10 | non-randomised studies | serious^a^ | not serious | not serious | not serious | none | 497 | 998 | - | MD **0.67 lower** (1.29 lower to 0.06 lower) | ⨁◯◯◯ Very low |  |
| **Length of Hospital Stay (Days)** | | | | | | | | | | | | |
| 9 | non-randomised studies | serious^a^ | not serious | not serious | not serious | strong association | 451 | 836 | - | MD **2.3 lower** (4.08 lower to 0.52 lower) | ⨁⨁◯◯ Low |  |
| **Mortality (Overall)** | | | | | | | | | | | | |
| 18 | non-randomised studies | serious^a^ | not serious | not serious | not serious | strong association | 35/3099 (1.1%) | 128/4440 (2.9%) | **RR 0.40** (0.27 to 0.60) | **17 fewer per 1,000** (from 21 fewer to 12 fewer) | ⨁⨁◯◯ Low |  |
| **Postoperative Cardiovascular Reintervention Rate** | | | | | | | | | | | | |
| 21 | non-randomised studies | serious^a^ | not serious | not serious | not serious | strong association | 129/2581 (5.0%) | 266/2375 (11.2%) | **RR 0.46** (0.34 to 0.63) | **60 fewer per 1,000** (from 74 fewer to 41 fewer) | ⨁⨁◯◯ Low |  |
| **Cardiopulmonary Bypass Time (CPBT)** | | | | | | | | | | | | |
| 15 | non-randomised studies | serious^a^ | not serious | not serious | not serious | none | 1484 | 1532 | - | MD **14.97 lower** (22.54 lower to 7.41 lower) | ⨁◯◯◯ Very low |  |
| **Aortic Cross-Clamp Time (Minutes)** | | | | | | | | | | | | |
| 17 | non-randomised studies | serious^a^ | not serious | not serious | not serious | none | 2218 | 2177 | - | MD **4.33 lower** (8.14 lower to 0.52 lower) | ⨁◯◯◯ Very low |  |
| **Low Cardiac Output (ECMO)** | | | | | | | | | | | | |
| 6 | non-randomised studies | not serious | not serious | not serious | not serious | strong association | 43/1098 (3.9%) | 44/835 (5.3%) | **RR 0.42** (0.28 to 0.64) | **31 fewer per 1,000** (from 38 fewer to 19 fewer) | ⨁⨁⨁◯ Moderate |  |
| **Acute Kidney Injury** | | | | | | | | | | | | |
| 5 | non-randomised studies | serious^d^ | not serious | not serious | not serious | strong association | 16/571 (2.8%) | 60/677 (8.9%) | **RR 0.29** (0.16 to 0.51) | **63 fewer per 1,000** (from 74 fewer to 43 fewer) | ⨁⨁◯◯ Low |  |
| **Postoperative Inotropic Support Duration (Hours)** | | | | | | | | | | | | |
| 4 | non-randomised studies | serious^a^ | very serious^b^ | not serious | serious^e^ | strong association | 152 | 483 | - | MD **17.64 lower** (53.42 lower to 18.13 higher) | ⨁◯◯◯ Very low |  |
| **Pleural Effusion** | | | | | | | | | | | | |
| 4 | non-randomised studies | serious^d^ | not serious | not serious | not serious | strong association | 9/161 (5.6%) | 68/495 (13.7%) | **RR 0.47** (0.24 to 0.92) | **73 fewer per 1,000** (from 104 fewer to 11 fewer) | ⨁⨁◯◯ Low |  |
| **Neurological Complication** | | | | | | | | | | | | |
| 4 | non-randomised studies | serious^d^ | not serious | not serious | not serious | none | 3/173 (1.7%) | 26/543 (4.8%) | **RR 0.60** (0.15 to 2.45) | **19 fewer per 1,000** (from 41 fewer to 69 more) | ⨁◯◯◯ Very low |  |
| **Chylothorax** | | | | | | | | | | | | |
| 3 | non-randomised studies | serious^d^ | not serious | not serious | not serious | strong association | 5/114 (4.4%) | 23/169 (13.6%) | **RR 0.34** (0.09 to 1.22) | **90 fewer per 1,000** (from 124 fewer to 30 more) | ⨁⨁◯◯ Low |  |
| **Pericardial Effusion** | | | | | | | | | | | | |
| 2 | non-randomised studies | very serious^f^ | not serious | not serious | not serious | strong association | 2/122 (1.6%) | 48/441 (10.9%) | **RR 0.22** (0.03 to 1.52) | **85 fewer per 1,000** (from 106 fewer to 57 more) | ⨁◯◯◯ Very low |  |

**CI:** confidence interval; **MD:** mean difference; **RR:** risk ratio

#### Explanations

a. A majority of studies had a moderate/serious risk of bias due to lack of control for confounding. This was, however, offset by the subgroup analysis that was performed for subgroup B.

b. I^2 > 75%

c. Very wide confidence interval.

d. A majority of studies had a moderate/serious risk of bias in this outcome due to lack of control for confounding.

e. Wide confidence interval.

f. Only studies rated as moderate/serious risk of bias were included in this outcome.
